# Machine Learning–Driven QSAR Modeling for pK_a_ Prediction of Ionizable Lipids in Lipid Nanoparticles for Hepatic Gene Silencing

**DOI:** 10.3390/ijms27094075

**Published:** 2026-05-01

**Authors:** Napat Kongtaworn, Borwornlak Toopradab, Duangjai Todsaporn, Poomrapee Tinpovong, Rada Thongsuebsaeng, Phornphimon Maitarad, Thanyada Rungrotmongkol

**Affiliations:** 1 Program in Bioinformatics and Computational Biology, College of Interdisciplinary and Integrative Studies, Chulalongkorn University, Bangkok 10330, Thailand; napat9079@gmail.com (N.K.); piyajaree@gmail.com (B.T.); poomrapee.tin@gmail.com (P.T.); 2 Center of Excellence in Structural and Computational Biology, Department of Biochemistry, Faculty of Science, Chulalongkorn University, Bangkok 10330, Thailand; fai.dt8@gmail.com (D.T.); rada.mnm27@gmail.com (R.T.); 3 Research Center of Nano Science and Technology, College of Science, Shanghai University, Shanghai 200444, China

**Keywords:** lipid nanoparticles, ionizable lipid design, pK_a_ prediction, machine learning QSAR, gene silencing

## Abstract

Liver cancer remains a significant global health burden, requiring the development of precise nucleic acid delivery systems. Lipid nanoparticles (LNPs) are leading candidates; however, their efficiency is governed by the pK_a_ of ionizable lipids, which dictates nanoparticle stability and endosomal escape. In this study, we employed a machine learning–driven quantitative structure–activity relationship framework to predict the pK_a_ of ionizable lipids derived from the DLin–KC2–DMA scaffold. Utilizing a dataset of 56 compounds, we compared Random Forest, Artificial Neural Network, and Extreme Gradient Boosting (XGB) models integrated with Permutation Importance (PI) for feature selection. The optimized PI–XGB model exhibited exceptional predictive accuracy (R^2^ = 0.970, R^2^_CV_ = 0.901, RMSE_test_ = 0.115) and robust generalization confirmed via external validation (RMSE_ext._ = 0.313). Mechanistic insights derived from SHapley Additive exPlanation analysis identified charge distribution, molecular topology, and polarity as critical determinants of lipid ionization. These results demonstrate the power of interpretable machine learning in elucidating molecular structure–property relationships, offering a robust computational strategy for the rational design of next–generation ionizable lipids to optimize LNP–mediated gene therapy for liver cancer.

## 1. Introduction

Over recent decades, survival outcomes for many diseases have steadily improved; however, liver disease remains a notable exception. As emphasized by The Lancet Commission [1], the global burden of liver disorders—including liver cancer, hepatitis, alcoholic liver disease, fatty liver disease, and hereditary conditions—continues to rise. Beyond causing structural and cellular damage, these diseases impair essential metabolic functions of the liver [2]. Among them, liver cancer is particularly severe, ranking as the sixth most common malignancy and the third leading cause of cancer–related mortality worldwide [3,4]. Current treatments—surgical resection, chemotherapy, and liver transplantation—are constrained by tumor stage, systemic toxicity, donor scarcity, and the risk of recurrence [5], underscoring the need for more selective and effective therapeutic strategies.

Hepatocellular carcinoma (HCC) remains one of the most lethal malignancies globally, characterized by its high heterogeneity and poor clinical outcomes. For patient with advanced HCC, the prognosis is particularly dismal, with a median overall survival of only 10–12 months and a 5–year survival rate of approximately 18% [1,2]. While traditional interventions—including surgical resection, liver transplantation, and cytotoxic chemotherapy—form the backbone of HCC management, their efficacy is often compromised by late–stage diagnosis and significant systemic toxicity [3]. The therapeutic landscape has recently evolved with the introduction of targeted therapies (e.g., Sorafenib, Lenvatinib) and immunotherapies, specifically immune checkpoint inhibitors like the Atezolizumab/Bevacizumab combination, which have shown superior survival benefits compared to traditional chemotherapy [6,7]. However, despite these advancements, challenges such as primary and acquired drug resistance, severe immune–related adverse events, and limited patient response rate persist as major hurdles in HCC treatment [7]. These limitations necessitate the development of more precise molecular strategies that can bypass systemic toxicity and directly modulate oncogenic drivers.

In this context, gene silencing via RNA interference (RNAi) emerges as a transformative alternative. RNAi–based therapeutics, such as small interfering RNA (siRNAs), offer a more targeted approach by specifically suppressing oncogenic pathways at the post–transcriptional level. Unlike small molecule inhibitors that require accessible binding pockets on proteins, RNAi can theoretically target any gene, including those previous considered “undruggable” [8,9]. Despite this potential, the clinical translation of RNAi is hampered by significant extracellular and intracellular barriers. Naked RNA molecules are inherently unstable, susceptible to nuclease–mediated degradation, and unable to across the anionic cellular membrane due to their high molecular weight and negative charge [10,11]. To overcome these, lipid nanoparticles (LNPs) have emerged as the gold–standard delivery platform. A typical LNP consists of cholesterol, phospholipids, PEG–lipids, and most critically ionizable lipids [12]. The acid dissociation constant (pK_a_) of these ionizable lipids is the key determinant of therapeutic success. Optimal LNPs must maintain a pK_a_ in the range of 6.0–7.0, ensuring they remain neutral at physiological pH (7.4) to minimize toxicity while becoming protonated within the acidic environment of the endosome (pH ≈ 5.0–6.0). This protonation triggers a transition from a lamellar to a hexagonal phase, promoting membrane destabilization and the subsequent release of the RNA cargo into the cytosol—a process known as endosomal escape [13]. As endosomal escape is recognized as the primary rate–limiting step in nucleic acid delivery, the rational design of ionizable lipids with precise pK_a_ values is essential. However, the experimental determination of pK_a_ is both costly and labor–intensive [14,15]. Consequently, there is an urgent need for computational frameworks, such as quantitative structure–activity relationship (QSAR) models driven by machine learning, to accelerate the discovery of novel ionizable lipids with optimized ionization behaviors for potent gene silencing in HCC therapy.

Computational methodologies, particularly QSAR and machine learning (ML), have gained traction as efficient alternatives for predicting lipid pK_a_. A validated QSAR model for 56 amine–containing heterolipids achieved strong predictive performance (internal R^2^ = 0.972; external predicted R^2^ ≈ 0.630) and demonstrated high concordance with experimental pK_a_ values [15]. Subsequent studies have expanded ML integration into LNP design, showing that lipid structure, ionization behavior, and local microenvironment collectively influence delivery efficiency following both intramuscular and intravenous administration [16,17,18]. Advanced computational frameworks combining electrostatic modeling and molecular simulations further improve apparent pK_a_ estimation within nanoparticle assemblies [19]. Artificial intelligence has also accelerated virtual lipid screening by identifying candidates with optimal ionization characteristics, thereby reducing reliance on combinatorial experimentation [20,21,22,23].

While the earlier study by Dhumal et al. [14] established foundational QSAR approaches for lipid pKa prediction, the present work extends this framework in several critical ways. Most importantly, we integrated multiple complementary descriptor selection methods (genetic function approximation; GFA, variance inflation factor; VIF, and permutation importance; PI) rather than relying on a single approach, enabling identification of mechanistically distinct descriptor sets that each capture different aspects of lipid ionization behavior. Furthermore, we employed ensemble machine learning algorithms (random forest; RF, artificial neural network; ANN, extreme gradient boosting; XGB) systematically compared across all descriptor selection strategies, providing comprehensive analysis of algorithm–descriptor combinations. Importantly, the addition of Shapley Additive explanation (SHAP) –based interpretability analysis in the current study provides unprecedented mechanistic insights into how individual descriptors govern lipid pK_a_, moving beyond pure predictive accuracy to elucidate casual structure–property relationships. Additionally, our external validation using structurally diverse lipids from independent literature sources [24] provides stronger evidence of model transferability and generalizability.

Recent advances in QSAR–ML have transformed pK_a_ prediction from traditional descriptor–based regression into data–driven, physically interpretable modeling. Interpretable deep–learning frameworks integrating SHAP analysis have improved prediction accuracy while revealing mechanistic insights into ionization equilibria [25]. Transfer–learning approaches have enhanced generalizability across chemically diverse datasets [26], while graph neural networks enriched with quantum–chemical features have enabled accurate prediction of protein residue pK_a_ values [27]. Parallel developments in LNP formulation highlight the growing role of ML–guided design, integrated databases, and high–throughput screening in identifying non–intuitive structure–property relationships [28,29,30,31]. Recent cutting–edge research has further emphasized the importance of structure–guided ionizable lipid design. Comprehensive investigations into messenger RNA (mRNA) and siRNA delivery have demonstrated that systematic variation in lipid ionization properties can substantially enhance therapeutic outcomes in both preclinical and clinical settings. These advances underscore the critical need for computational frameworks that can accurately predict and rationalize lipid ionization behavior.

These advances are exemplified by recent studies employing AI–assisted ionizable lipid prediction and design. Xu et al. [32] reported significant advances in structure–guided lipid design for enhanced mRNA delivery. Emerging research has demonstrated the importance of ionization properties in optimizing nucleic acid delivery across diverse tissues [33]. Additionally, highlighted the computational approaches and machine learning strategies for accelerating ionizable lipid discovery [34]. These cutting–edge developments underscore the critical importance of computational tools, such as the QSAR framework presented here, for rational ionizable lipid design.

**Scheme 1:**
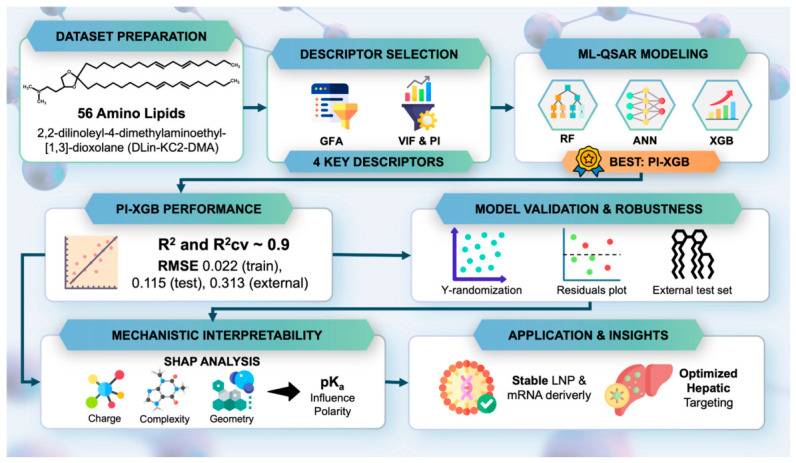
Overview of the machine learning–driven QSAR workflow for predicting the pK_a_ of ionizable amino lipids used in lipid nanoparticle–mediated gene delivery.

In this study, we applied a QSAR–ML framework to predict the pK_a_ values of ionizable amino lipids derived from 2,2–dilinoleyl–4–dimethylaminoethyl–[1,3]–dioxolane (DLin–KC2–DMA), a benchmark lipid used in LNP formulations for siRNA delivery. Experimental pK_a_ values reported by Jayaraman et al. [35] were used to construct a dataset consisting of 56 lipid derivatives. In these molecules, the hydrophobic dilinoleyl tail was conserved to maintain membrane fluidity and fusogenic properties, while systematic modifications to the ionizable head group were introduced to probe structural determinants governing pK_a_. As illustrated in Scheme 1, molecular descriptors were first calculated and filtered using feature selection strategies, including GFA, VIF, and PI, to identify key descriptors relevant to lipid ionization behavior. Machine learning models were then developed using RF, ANN, and XGB algorithms to establish quantitative relationships between molecular structure and pK_a_. Among the evaluated models, the PI–XGB model demonstrated the best predictive performance, with strong correlation (R^2^ and R^2^CV ≈ 0.9) and low prediction errors. Model robustness was further evaluated using Y–randomization tests, residual analysis, and an independent external set [36]. Finally, SHAP–based interpretability analysis was employed to elucidate how physicochemical descriptors related to charge distribution, molecular complexity, and geometric features influence lipid pK_a_. Together, these results provide mechanistic insights into the structural factors governing ionizable lipid behavior and offer a predictive framework to support the rational design of next–generation LNP systems for efficient hepatic gene silencing.

## 2. Results

### 2.1. Feature Analysis

Reliable QSAR model development requires appropriate descriptor reduction to avoid overfitting and ensure robust statistical performance. In this study, descriptor selection followed the commonly applied “1–in–10 rule,” which recommends that the number of descriptors in a QSAR model should not exceed one–tenth of the number of training compounds [37,38]. Given the 45 compounds in the training set, the descriptor number was limited to four variables. Accordingly, the initial pool of 47 descriptors was reduced to a compact set of 4 for model construction, while the independent test set was reserved for external validation.

#### 2.1.1. Descriptor Selection Using GFA and VIF

**Figure 1:**
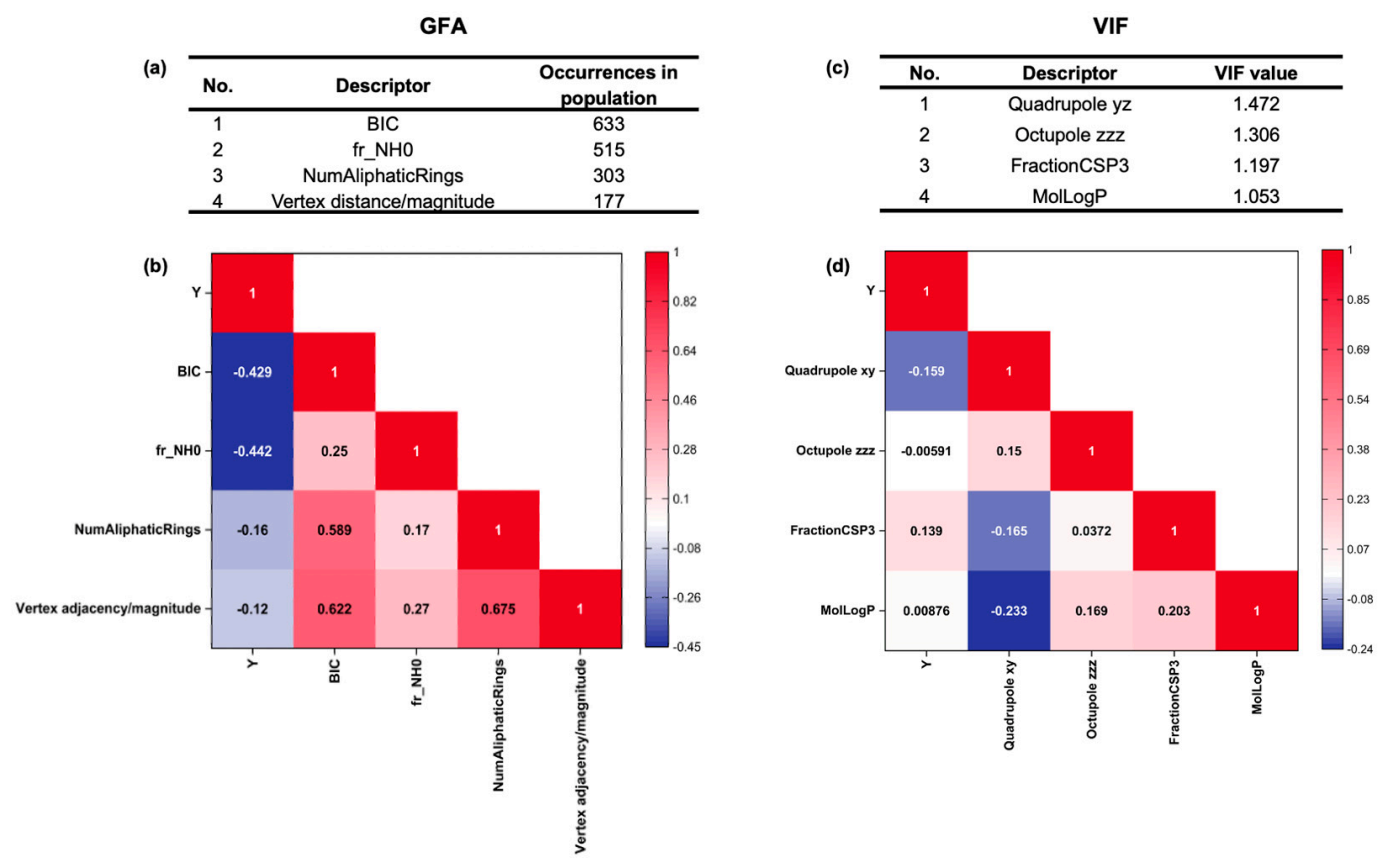
Descriptor selection and multicollinearity analysis. (**a**) Occurrence frequency of descriptors identified by the GFA method during model population generation. (**b**) Correlation matrix of descriptors selected by GFA. (**c**) VIF values of descriptors retained after multicollinearity filtering. (**d**) Correlation matrix of descriptors after VIF–based selection.

The GFA method was first applied to identify descriptors strongly associated with pK_a_ variation. Based on occurrence frequency in the generated model populations, four descriptors—bond information content (BIC), fr_NH0, NumAliphaticRings, and Vertex distance/magnitude—were selected (Figure 1a). Among them, BIC and fr_NH0 showed the highest occurrence frequencies, suggesting their strong contribution to model performance.

The correlation matrix of the GFA–selected descriptors (Figure 1b) indicates moderate correlations among several descriptors. In particular, NumAliphaticRings and Vertex distance/magnitude exhibited a relatively high positive correlation (r = 0.675), while BIC also showed moderate correlations with these variables. Although these correlations did not exceed typical multicollinearity thresholds, further evaluation was performed to ensure descriptor independence.

To refine the descriptor set, VIF analysis was conducted. After VIF filtering, four descriptors—Quadrupole xy, Octupole zzz, FractionCSP3, and MolLogP—were retained (Figure 1c). All descriptors exhibited low VIF values (1.053–1.472), confirming the absence of significant multicollinearity.

The correlation matrix of the final descriptor set (Figure 1d) shows low inter–descriptor correlations, indicating that each descriptor contributes largely independent information to the model. MolLogP and FractionCSP3, representing lipophilicity and molecular saturation, respectively, capture complementary physicochemical features. Meanwhile, the electrostatic descriptors Quadrupole xy and Octupole zzz encode distinct aspects of molecular charge distribution. Together, these descriptors provide a balanced representation of structural and electronic properties relevant to lipid ionization behavior.

#### 2.1.2. Descriptor Selection Using PI

**Figure 2:**
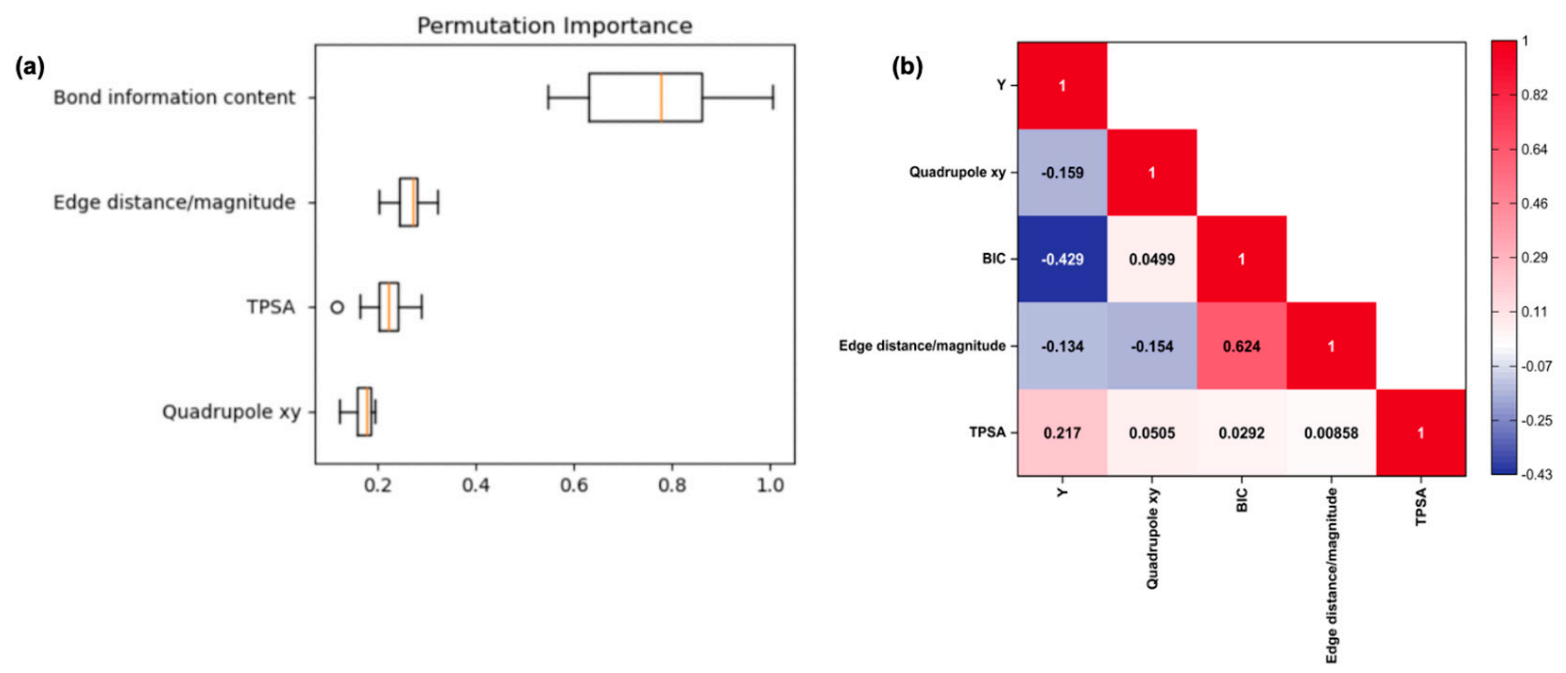
Descriptor importance and correlation analysis of the PI–selected variables. (**a**) PI ranking of the four selected descriptors. (**b**) Correlation matrix of the PI–selected descriptors.

PI analysis was performed to identify the descriptors that contribute most strongly to the predictive performance of the optimized QSAR–ML model. The results are summarized in Figure 2, which presents both the relative importance of the descriptors and their correlation matrix. PI analysis identified four key descriptors—Quadrupole xy, BIC, Edge distance/magnitude, and topological polar surface area (TPSA) as the most influential variables governing the predicted response (Figure 2a).

Among these descriptors, BIC exhibited the highest importance score, indicating that molecular bonding complexity plays a dominant role in determining the modeled endpoint. Edge distance/magnitude showed moderate importance, reflecting contributions from molecular topology and connectivity. In contrast, TPSA and Quadrupole xy showed lower but meaningful values, suggesting that molecular polarity and electrostatic charge distribution also contribute to predicting the target property.

The correlation matrix of the PI–selected descriptors (Figure 2b) shows generally low inter–descriptor correlations, indicating minimal redundancy among the selected variables. The highest correlation was observed between BIC and Edge distance/magnitude (r = 0.624), suggesting partial overlap in topological information while remaining below typical multicollinearity thresholds. TPSA and Quadrupole xy exhibited weak correlations with the other descriptors, indicating that polarity and electrostatic descriptors provide complementary information to the model. Overall, the PI analysis confirms that the final descriptor set captures complementary structural, topological, and electrostatic features, providing a balanced representation of the molecular determinants governing lipid ionization behavior.

To characterize the structural and physicochemical properties of the studied compounds, a set of key molecular descriptors was calculated using RDKit. These descriptors, encompassing topological, electronic, and geometric features, are summarized with their respective definition in Table 1.

### 2.2. QSAR–ML Model Performance

**Figure 3:**
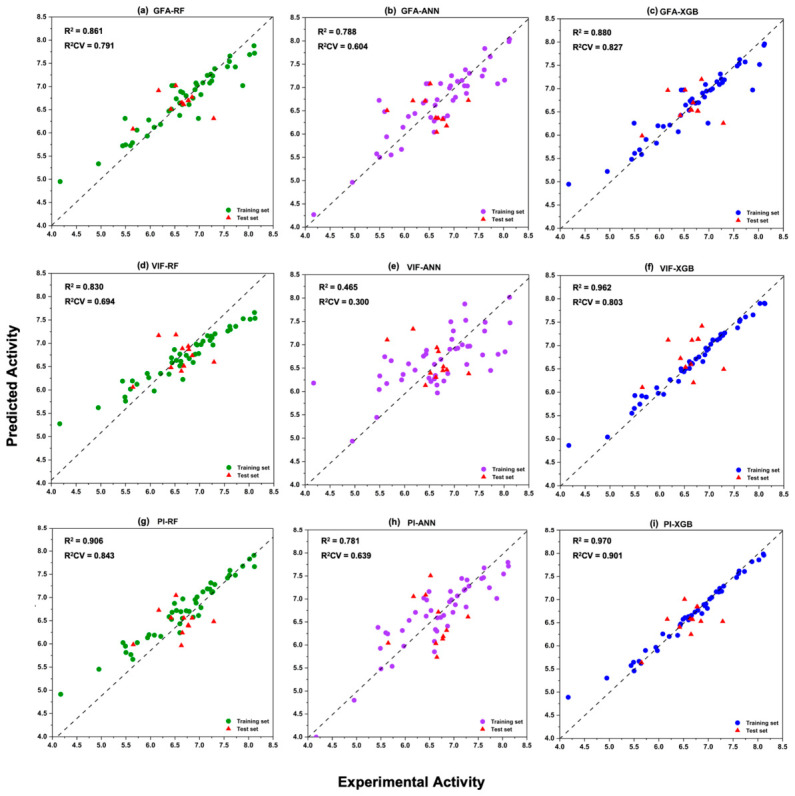
Scatter plots of experimental versus predicted pKa values for QSAR–ML models developed using different descriptor selection methods and machine learning algorithms with statistical performance (R^2^_train_ and R^2^CV). (**a**–**c**) Models based on GFA–selected descriptors: (**a**) RF, (**b**) ANN, and (**c**) XGB. (**d**–**f**) Models based on VIF–selected descriptors: (**d**) RF, (**e**) ANN, and (**f**) XGB. (**g**–**i**) Models based on PI–selected descriptors: (**g**) RF, (**h**) ANN, and (**i**) XGB. Green, purple, and blue circles represent the training set of RF, ANN, and XGB, respectively. Red triangles represent the test set.

The predictive performance of the developed QSAR models was evaluated using three machine learning algorithms (RF, ANN, and XGB) combined with three descriptor selection strategies (GFA, VIF, and PI). The agreement between experimental and predicted pK_a_ values is illustrated in Figure 3, while the corresponding statistical parameters are summarized in Table 2. Detailed prediction results for all 56 compounds are provided in Tables S3–S5 for the GFA–, VIF–, and PI–selected descriptor sets, respectively.

As shown in Figure 3, the predictive performance of the models varies depending on both the descriptor selection method and the learning algorithm. For the GFA–based models (Figure 3a–c), the RF and XGB models show reasonable agreement between experimental and predicted values, with most data points clustering near the diagonal line. In contrast, the ANN model (Figure 3b) shows greater scatter, particularly for the test set compounds, indicating lower prediction stability.

For the VIF–selected descriptors (Figure 3d–f), similar trends are observed. The VIF–XGB model (Figure 3f) shows improved clustering of predicted values along the diagonal line compared with RF and ANN models. The VIF–ANN model (Figure 3e) again exhibits larger deviations from the diagonal line, suggesting reduced predictive reliability.

The best predictive performance was obtained using PI–selected descriptors combined with the XGB algorithm, as illustrated in Figure 3i. In this model, both training and test data points align closely with the diagonal line, indicating excellent agreement between experimental and predicted pK_a_ values across the entire dataset. This observation is consistent with the statistical metrics reported in Table 2, where the PI–XGB model achieved the highest R^2^ (0.970) and R^2^CV (0.901) along with the lowest prediction errors (RMSE_train_ = 0.022 and RMSE_test_ = 0.115).

The PI–RF model (Figure 3g) also demonstrates good predictive performance, although the dispersion of data points is slightly larger than that observed for PI–XGB. In contrast, ANN–based models (Figure 3b,e,h) consistently show greater variability in predicted values, which may reflect the sensitivity of neural networks to limited dataset size and descriptor configuration.

The comparative analysis of descriptor selection methods and machine learning algorithms reveals important insights into model development strategies. While GFA–based selection identified descriptors with strong predictive frequency, its correlation matrix showed moderate inter–descriptor correlations. In contrast, VIF–based selection yielded descriptors with minimal multicollinearity but showed variable performance depending on the learning algorithm. The PI–based approach, which prioritizes descriptors by their direct contribution to model prediction, produced the most balanced and stable descriptor set when combined with XGB. This synergy likely arises from the PI approach identifying features with genuine mechanistic relevance to lipid ionization, which the XGB algorithm can effectively integrate through its gradient–boosting ensemble strategy.

Collectively, the PI–XGB model exhibited the best predictive performance for pK_a_ estimation, demonstrating superior accuracy and robustness. These results highlight the advantage of integrating informative descriptor selection with ensemble learning algorithms to develop reliable and interpretable QSAR–ML models.

### 2.3. QSAR–ML Models Validation

#### 2.3.1. External Set Prediction

**Figure 4:**
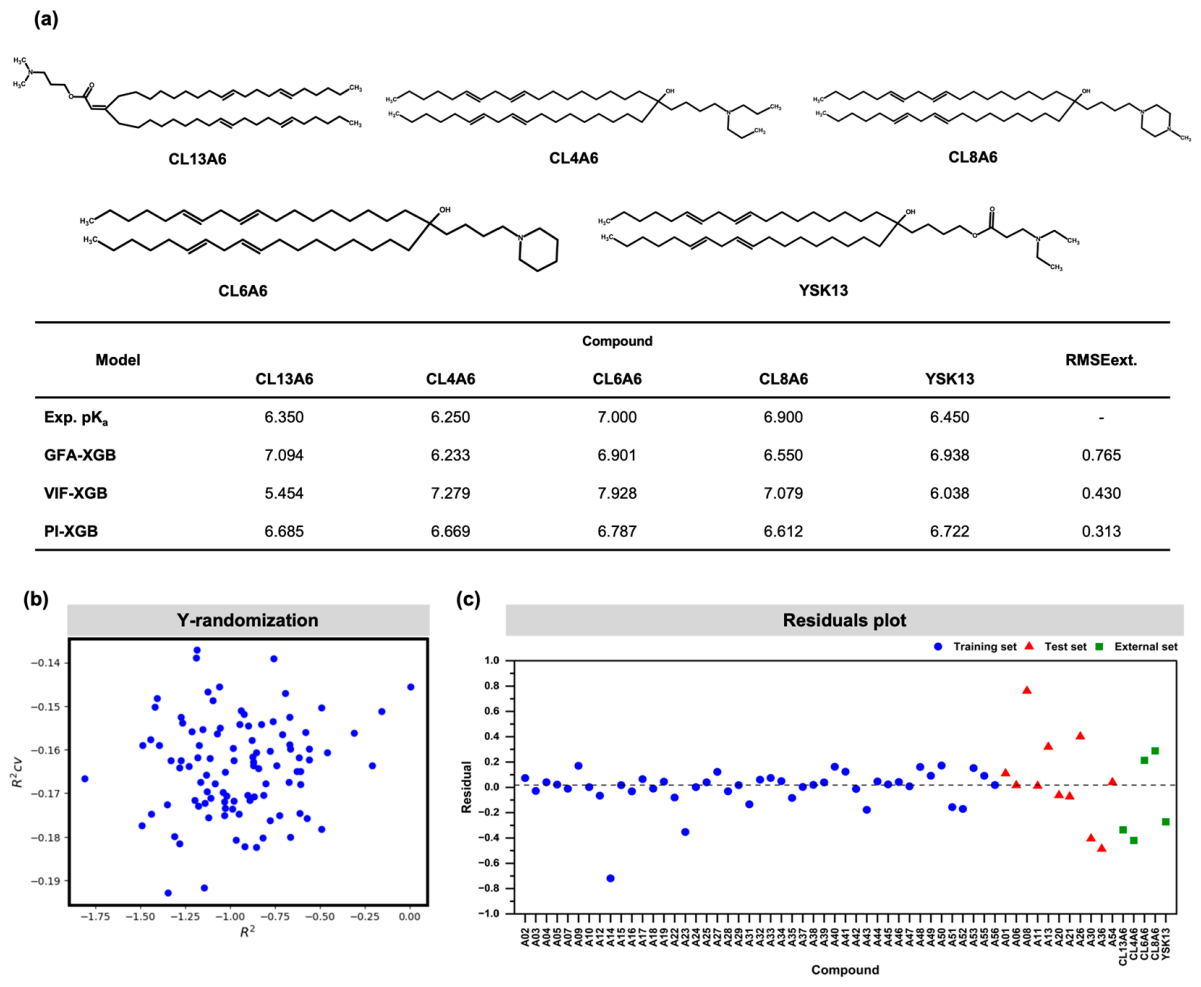
Validation and robustness analysis of the QSAR–ML pK_a_ prediction models. (**a**) Chemical structures of the external validation compounds and their corresponding experimental and predicted pK_a_ values. (**b**) Y–randomization test for the PI–XGB model. (**c**) Residual distribution for training, test, and external datasets of the PI–XGB model.

To further evaluate the predictive reliability and generalizability of the developed QSAR–ML models, external validation was performed using five structurally diverse ionizable lipids collected from independent literature sources [36]. These compounds were not included in either the training or test datasets and therefore provide an unbiased assessment of model performance. The chemical structures and predicted pKa values of the external compounds are summarized in Figure 4a, while the calculated descriptors are provided in Table S6.

Among the evaluated models, the PI–XGB model exhibited the best external predictive performance, yielding the lowest RMSE_ext._ (0.313) compared with VIF–XGB (0.430) and GFA–XGB (0.765). The relatively small prediction errors [38,39,40] observed across the training, test, and external datasets indicate that the PI–XGB model maintains strong predictive capability when applied to previously unseen compounds.

Inspection of the individual predictions further confirms the model’s reliability. For example, a compound with an experimental pK_a_ of 6.900 was predicted as 6.612 by the PI–XGB model, corresponding to a deviation of only 0.288 pK_a_ units. In contrast, the GFA–XGB model showed larger deviations for several external compounds, consistent with its higher external prediction error. These results demonstrate that the PI–XGB model provides the most accurate and transferable prediction for structurally related ionizable lipids.

#### 2.3.2. Y–Randomization and Residual Analysis

Additional validation was performed using Y–randomization and residual analysis to evaluate the robustness of the optimized PI–XGB model. As shown in Figure 4b, the Y–randomization test produced significantly lower R^2^ and Q^2^ values for the randomized models than for the original model, indicating that the observed predictive performance is not due to chance correlation.

The residual distribution plot (Figure 4c) further confirms model reliability. Residuals for the training, test, and external datasets are randomly distributed around zero, with no systematic patterns, suggesting the absence of structural bias or heteroscedasticity. Most residuals fall within a narrow range, indicating consistent prediction accuracy across the dataset.

Overall, the combined results from external validation, Y–randomization, and residual analysis demonstrate that the PI–XGB model is robust, statistically reliable, and capable of predicting the pK_a_ values of ionizable amino lipids beyond the training domain.

#### 2.3.3. SHAP Analysis

**Figure 5:**
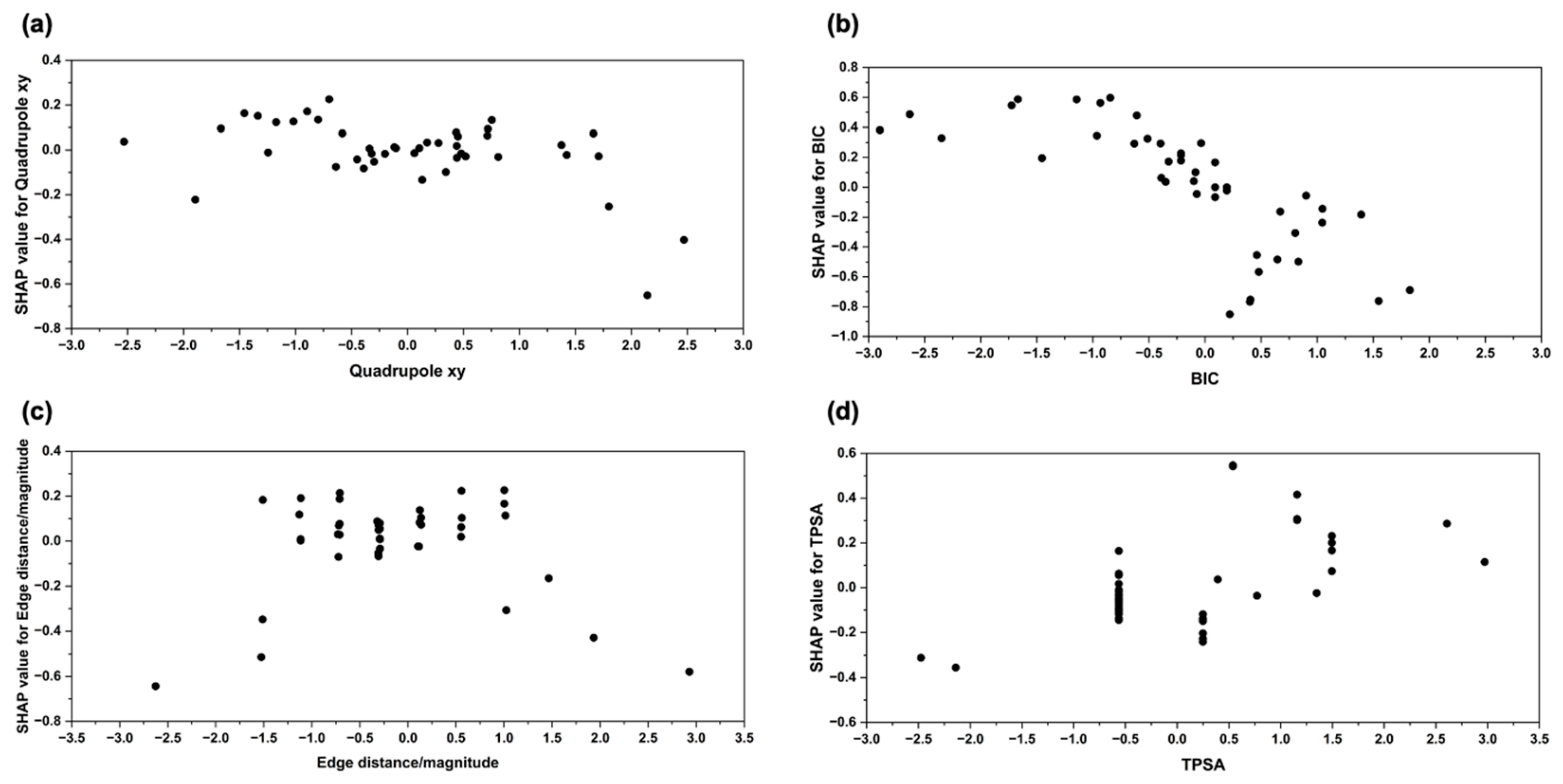
SHAP dependence plots illustrating the influence of key molecular descriptors on the predicted pK_a_ values of ionizable lipids. (**a**) Quadrupole xy, (**b**) BIC, (**c**) Edge distance/magnitude, and (**d**) TPSA. Each point represents an individual lipid molecule, where the SHAP value indicates the magnitude and direction of the descriptor’s contribution to the model prediction.

To further interpret the QSAR–ML model, SHAP analysis was performed to quantify the contribution of individual molecular descriptors to the predicted pK_a_ values. SHAP values provide a measure of how each descriptor influences the model prediction, with positive and negative values indicating contributions that increase or decrease the predicted response, respectively. The SHAP dependence plots for the four key descriptors are shown in Figure 5.

As illustrated in Figure 5a, the Quadrupole xy descriptor, which describes the anisotropic distribution of molecular charge in the xy–plane, shows a positive relationship with the SHAP values. This suggests that increased charge asymmetry contributes positively to the predicted pK_a_, likely reflecting the role of electrostatic interactions in lipid ionization behavior.

The BIC descriptor exhibits a negative trend with increasing SHAP values (Figure 5b). This indicates that greater molecular complexity and bonding diversity tend to reduce the predicted pK_a_ values. Simpler lipid structures with lower BIC values, therefore, appear to favor the physicochemical characteristics required for effective lipid nanoparticle assembly.

The Edge distance/magnitude descriptor, which captures aspects of molecular topology and spatial arrangement, displays a nonlinear relationship with SHAP values (Figure 5c). Only specific geometric configurations contribute positively to the prediction, suggesting that optimal spatial organization of lipid fragments is important for achieving favorable molecular packing within lipid nanoparticles.

Finally, the TPSA descriptor (Figure 5d) reflects a molecule’s polarity and hydrogen–bonding capacity. The SHAP results indicate that moderate TPSA values contribute more favorably to the predicted response, whereas extremely low or high TPSA values tend to have negative effects. This observation suggests that a balanced polarity is required to maintain both membrane compatibility and effective interactions with nucleic acids. Therefore, the SHAP analysis highlights the combined influence of electrostatic properties, molecular topology, structural complexity, and polarity on lipid ionization behavior. These insights provide mechanistic guidance for the rational design of ionizable lipids with optimized physicochemical properties for lipid nanoparticle–mediated gene delivery.

Overall, this study establishes a machine learning–based QSAR framework for predicting the pK_a_ values of ionizable amino lipids relevant to lipid nanoparticle (LNP) systems. By integrating descriptor selection strategies (GFA, VIF, and PI) with multiple machine learning algorithms, the PI–XGB model was identified as the most accurate and robust predictive model. Comprehensive validation, including cross–validation, external dataset prediction, Y–randomization, and residual analysis, confirmed the reliability and generalizability of the developed model. Furthermore, SHAP analysis provided mechanistic insights into how key molecular descriptors—such as charge distribution, molecular topology, structural complexity, and polarity—influence lipid ionization behavior. These findings highlight the value of interpretable machine learning approaches for uncovering structure–property relationships and provide practical guidance for the rational design of ionizable lipids with optimized physicochemical properties for LNP–mediated gene delivery.

The PI–XGB model achieved the highest predictive performance (R^2^ = 0.970, R^2^CV = 0.901, RMSE_test_ = 0.115) and the lowest external prediction error (RMSE_ext._ = 0.313), indicating superior generalizability. It also exhibited consistent performance across datasets, suggesting reduced overfitting. From a methodological perspective, XGB effectively captures nonlinear relationships and feature interactions, while PI prioritizes descriptors based on direct predictive contribution. This synergy provides optimal performance. Therefore, PI–XGB was selected as the most robust and reliable model for external validation.

## 3. Materials and Methods

### 3.1. Data Sources and Property Collections

A dataset comprising 56 structurally diverse ionizable amino lipids was used to develop ML–based QSAR models for pK_a_ prediction. The experimental pK_a_ values were obtained from the study reported by Jayaraman et al. [35], and the corresponding chemical structures and measured pK_a_ values are summarized in Table 3. In that work, the pK_a_ values were determined using the 2–(p–toluidino)–6–naphthalene sulfonic acid (TNS) fluorescence assay, which monitors changes in the surface charge of lipid nanoparticles (LNPs) as a function of pH. This assay provides a sensitive measure of lipid ionization behavior, a key physicochemical parameter that influences the performance of ionizable lipids in nucleic acid delivery systems.

Three–dimensional (3D) molecular structures of all lipids were constructed using Materials Studio 8.0 [40]. Molecular descriptors were subsequently calculated using the QSAR module in Materials Studio together with the RDKit descriptor library [41]. A total of 47 molecular descriptors were generated, representing diverse physicochemical properties including spatial, electronic, thermodynamic, topological, electrotopological (E–state), fragment–based, and geometric characteristics. These descriptors were used as independent variables to establish quantitative relationships between molecular structure and the experimentally measured pK_a_ values. The complete list of descriptors and their Pearson correlation coefficients is provided in Table S1 and Figure S1.

To ensure representative sampling of the chemical space, the dataset was divided into training and test sets using the Kennard–Stone (KS) algorithm, implemented in Python version 3.14.4 via the Kennard–Stone package [42]. This distance–based selection method distributes compounds evenly in descriptor space to maximize structural diversity. As a result, 45 compounds (80%) were assigned to the training set for model development, while the remaining 11 compounds (20%) were reserved as an external test set for independent model evaluation.

The curated descriptor matrix and dataset partition were subsequently used for feature selection and model development. Descriptor reduction was performed using GFA, VIF analysis, and PI to minimize multicollinearity and identify the most informative structural descriptors associated with lipid pK_a_. The selected descriptors were then used to construct QSAR–ML models using the RF, ANN, and XGB algorithms. Model robustness and predictive performance were evaluated through k–fold cross–validation, Y–randomization tests, and external validation, as described in the following sections.

### 3.2. Data Sources and Property Collections

Prior to model development, descriptor reduction was performed to improve model stability and minimize redundancy within the descriptor matrix. The initial descriptor pool consisted of 47 molecular descriptors representing structural, topological, electronic, and physicochemical properties of the ionizable amino lipids. Because the number of descriptors was comparable to the dataset size (56 compounds), a feature selection strategy was required to avoid overfitting and ensure reliable QSAR–ML model development [43,44].

A multi–stage feature selection procedure was implemented to identify the most informative descriptors. First, GFA was applied to identify descriptor combinations with strong predictive relevance to the experimental pK_a_ values. GFA uses evolutionary algorithms to iteratively select descriptor subsets that optimize statistical model fitness while maintaining chemical interpretability. Next, the VIF analysis was used to evaluate multicollinearity among the selected descriptors. Descriptors with VIF values exceeding the commonly accepted threshold were removed to ensure that the remaining variables were not strongly linearly correlated. This step improves the stability and interpretability of subsequent models by reducing redundancy within the descriptor set. In addition, PI analysis was performed to quantify the contribution of each descriptor to model prediction. In this approach, descriptor values are randomly permuted, and the resulting decrease in model performance is measured, enabling identification of descriptors with the greatest influence on predictive accuracy.

Finally, a Pearson correlation analysis was conducted to further examine pairwise relationships among descriptors and to eliminate highly correlated variables. The combined application of GFA, VIF filtering, PI analysis, and correlation analysis yielded a refined subset of descriptors that captured the key structural determinants of lipid ionization behavior while maintaining a compact descriptor space suitable for machine learning modeling.

These selected descriptors were subsequently used as input variables for the development of QSAR–ML models using RF, ANN, and XGB algorithms, as described in the following section.

#### 3.2.1. Genetic Function Approximation

GFA was employed as an initial method for selecting descriptors associated with lipid pK_a_ variation. GFA is an evolutionary algorithm that generates multiple regression models using different combinations of descriptors and evaluates their statistical performance using metrics such as the coefficient of determination (R^2^) and mean squared error. During evolution, descriptors that frequently appear in high–performing models are considered more relevant to the target property. This strategy allows efficient exploration of the descriptor space and facilitates identification of descriptor subsets with strong predictive relevance. The descriptors retained through GFA analysis served as candidate variables for subsequent multicollinearity filtering and machine learning model development [45].

#### 3.2.2. Variance Inflation Factor and Correlation Analysis

To reduce redundancy among descriptors, the VIF was used to assess multicollinearity within the selected descriptor set. VIF quantifies the degree to which the variance of a regression coefficient is inflated due to linear correlation with other descriptors. Descriptors with VIF values greater than 10 were considered highly collinear and were removed iteratively. VIF values were recalculated after each removal step until all remaining descriptors satisfied the threshold criteria [46].

Following VIF filtering, Pearson correlation analysis was performed to further examine pairwise relationships among descriptors. Pearson correlation coefficients range from –1 to +1, representing perfect negative and positive linear relationships, respectively. Descriptor pairs with absolute correlation coefficients |r| > 0.8 were considered strongly correlated [47,48]. From each correlated pair, the descriptor with lower physicochemical interpretability or weaker relevance to lipid ionization properties was removed. This procedure ensured that the final descriptor pool retained chemically meaningful information while minimizing redundancy.

#### 3.2.3. Permutation Importance

PI analysis was subsequently performed to evaluate the relative contribution of each descriptor to model prediction. In this approach, the values of a given descriptor are randomly permuted while all other descriptors remain unchanged, and the resulting change in model performance is measured. A significant decrease in predictive accuracy or an increase in prediction error indicates that the descriptor plays an important role in model prediction.

PI analysis was applied after statistical filtering to prioritize descriptors that contribute most strongly to pK_a_ prediction. The highly influential descriptors were retained for subsequent machine learning model construction. This procedure enabled the identification of a compact, informative descriptor set that captures the key structural determinants governing ionizable lipid behavior [49].

### 3.3. QSAR Model Development

Quantitative structure–activity relationship (QSAR) modeling was performed to establish relationships between the structural features of ionizable amino lipids and their experimentally measured pK_a_ values. Each compound was represented by a set of molecular descriptors derived from its chemical structure, including electronic, steric, topological, and physicochemical parameters. The selected descriptors obtained from the feature selection procedure described above were used as input variables for model construction.

Three machine learning algorithms were applied to develop predictive QSAR–ML models: RF, ANN, and XGB. RF models were constructed using an ensemble of decision trees trained on bootstrap samples of the training data, enabling robust modeling of nonlinear relationships between molecular descriptors and pK_a_ values [50,51]. ANN models were implemented as multilayer feed–forward networks capable of capturing complex nonlinear dependencies within the descriptor space. XGB models were developed using gradient–boosted decision trees, where trees are trained sequentially to minimize residual prediction errors, thereby improving predictive accuracy and generalization [52].

All models were implemented using the scikit–learn Python library. Model hyperparameters were optimized using a grid search with cross–validation to identify parameter combinations that yielded the best predictive performance. The explored hyperparameter ranges and the optimized values for the RF, ANN, and XGB models are summarized in Table S2. Model performance was evaluated using both the training set and the external test set generated by the Kennard–Stone algorithm. The final QSAR models were selected based on their predictive accuracy and statistical robustness [53,54], as assessed by the validation procedures described in the following section.

Y–randomization tests (response permutation testing) were conducted to verify that the observed correlation between molecular descriptors and experimental pKₐ values was not due to chance. In this procedure, the response variable (pK_a_) was randomly permuted while maintaining the descriptor matrix and model architecture. A series of randomized models was generated, and their R^2^ and Q^2^ values were compared with those of the original model. Significantly lower performance obtained for the randomized models confirmed that the developed QSAR models captured genuine structure–property relationships rather than random correlations [55].

Residual analysis was performed to evaluate prediction errors and detect potential systematic deviations. Residuals, defined as the differences between experimental and predicted pK_a_ values, were plotted against predicted values. For a reliable model, residuals should be randomly distributed around zero, with no observable trends, indicating the absence of systematic bias or heteroscedasticity [56].

To further assess model generalizability, external validation was carried out using an independent dataset of cationic lipids reported by Sato et al. [36]. Predictive performance was evaluated using the external root–mean–square error (RMSE_ext._). Consistent prediction accuracy between the test and external datasets indicates good model robustness and applicability for predicting pK_a_ values of newly designed ionizable lipids.

## 4. Conclusions

In this study, a machine learning–based QSAR framework was developed to predict the pK_a_ values of ionizable amino lipids derived from the DLin–KC2–DMA scaffold, which are widely used in lipid nanoparticle (LNP) systems for nucleic acid delivery. By integrating descriptor selection methods (GFA, VIF, and PI) with multiple machine learning algorithms, the PI–XGB model was identified as the optimal predictive model, demonstrating high accuracy and robustness (R^2^ = 0.970, R^2^_CV_ = 0.901, RMSE_train_ = 0.022, RMSE_test_ = 0.115, and RMSE_ext._ = 0.313). Validation via Y–randomization, residual analysis, and external–dataset prediction confirmed that the model captures meaningful structure–property relationships and exhibits strong generalization. Importantly, SHAP analysis provided mechanistic insights into the molecular determinants of lipid ionization behavior, highlighting the roles of charge distribution, molecular topology, structural complexity, and polarity. These findings suggest that an appropriate balance of electrostatic properties and molecular architecture is essential for optimizing ionizable lipids in LNP formulations.

Overall, this work demonstrates that interpretable machine learning approaches can effectively support the rational design of ionizable lipids with tailored physicochemical properties. The proposed framework provides a useful computational tool for accelerating the development of LNP–based gene delivery systems and may help improve therapeutic strategies for liver cancer and other diseases involving nucleic acid therapeutics.

## Figures and Tables

**Table 1:** Definitions and physical significance of the key RDKit molecular descriptors used in this study.

Descriptor	Definition
BIC	Measure molecular complexity based on bonding patterns and atom diversity.
fr_NH0	Counts tertiary amines (Nitrogen atoms with zero attached Hydrogens)
NumAliphaticRings	Total count of non–aromatic (saturated) rings in the molecule.
Vertex distance	Quantifies 3D molecular shape and spatial extent via atom vertices.
Quadrupole xy	Measures electronic charge asymmetry in the *xy*–plane.
Edge distance	Reflects connectivity and distances along the molecular bond paths.
TPSA	Surface area of polar atoms (O, N); indicates H–bonding and permeability.
FractionCSP3	Ratio of *sp*^3^ carbons to total carbons; measures saturation and flexibility.

**Table 2:** Statistical performance of RF, ANN, and XGB QSAR–ML models developed using descriptor sets selected by GFA, VIF, and PI models.

Model	R^2^	R^2^CV	RMSE_train_	RMSE_test_
GFA–RF	0.861	0.791	0.099	0.180
GFA–ANN	0.788	0.604	0.182	1.194
GFA–XGB	0.880	0.827	0.086	0.208
VIF–RF	0.830	0.694	0.121	0.206
VIF–ANN	0.465	0.300	0.336	2.023
VIF–XGB	0.962	0.803	0.027	0.206
PI–RF	0.906	0.843	0.067	0.215
PI–ANN	0.781	0.639	0.157	0.459
PI–XGB	0.970	0.901	0.022	0.115

**Table 3:** Representative structures of the ionizable lipid dataset with their respective pK_a_ values used for QSAR−ML model development.

Code	Structure	pK_a_	Code	Structure	pK_a_
A01 *	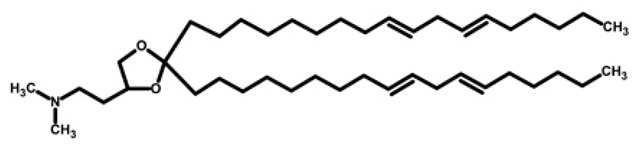	6.680	A09	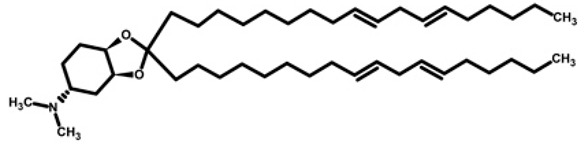	6.980
A02	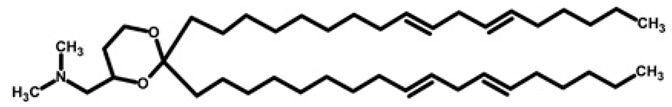	5.970	A10	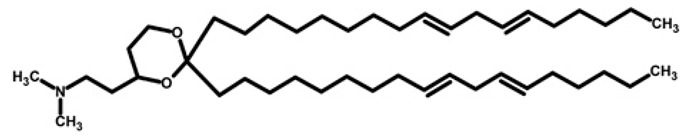	6.730
A03	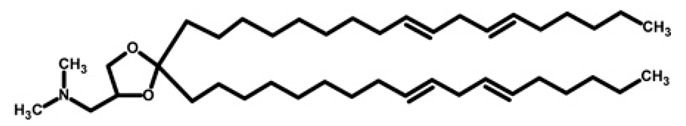	5.940	A11 *	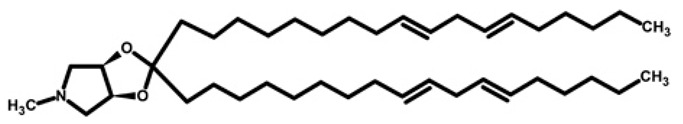	5.650
A04	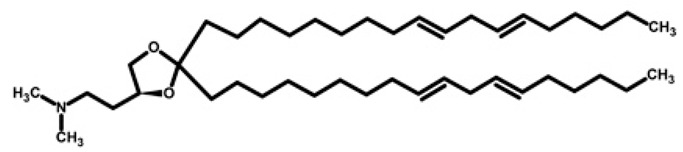	6.650	A12	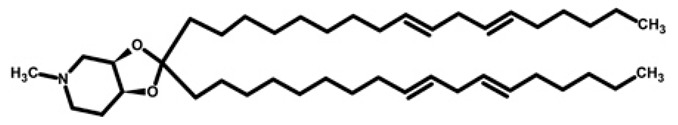	5.600
A05	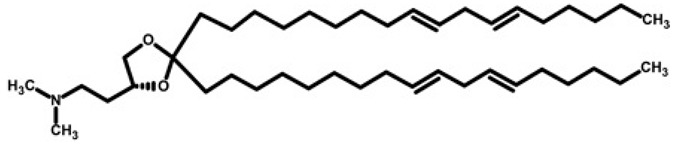	6.790	A13	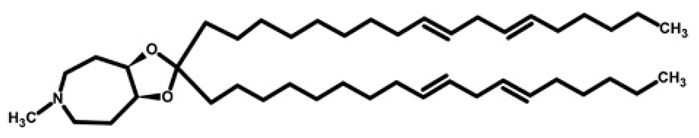	6.850
A06 *	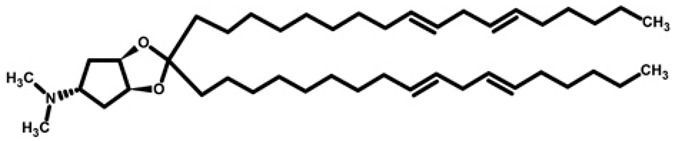	6.420	A14	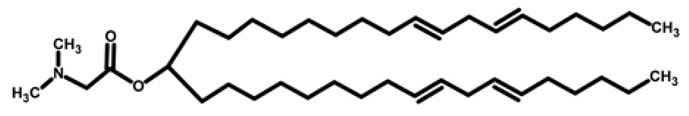	4.170
A07	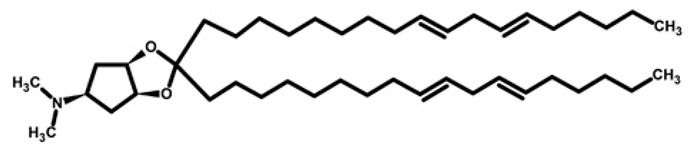	6.430	A15	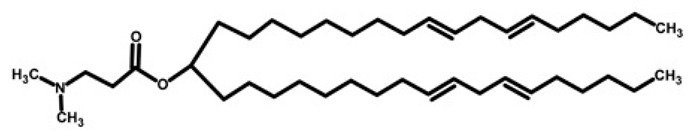	5.640
A08 *	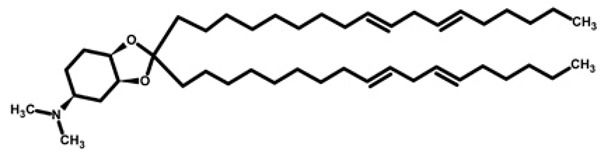	7.290	A16	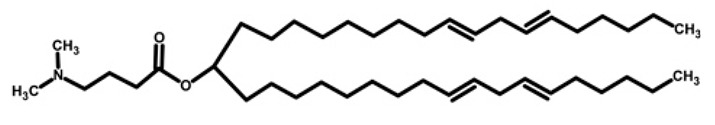	6.440
A17	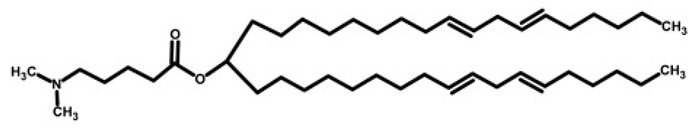	6.930	A37	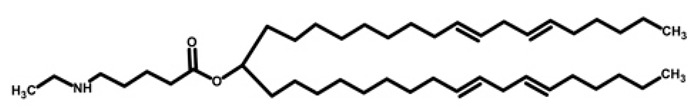	7.620
A18	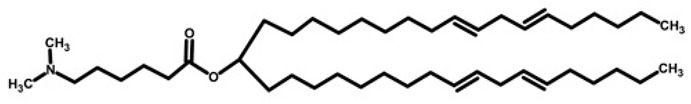	7.160	A38	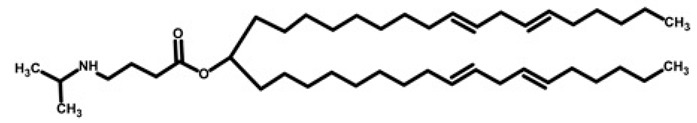	7.310
A19	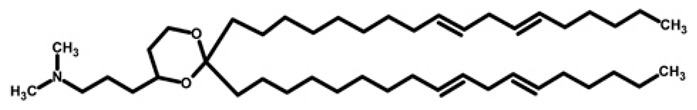	6.950	A39	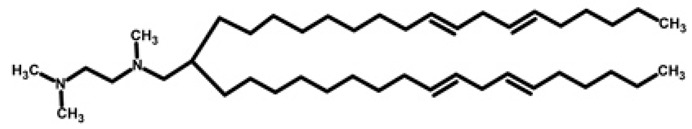	6.620
A20 *	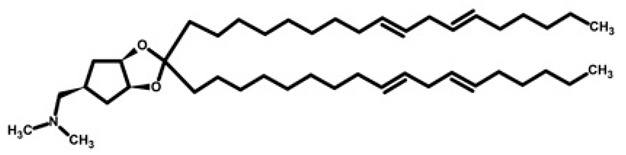	6.780	A40	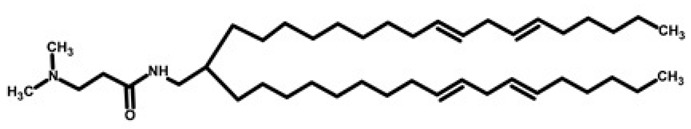	8.020
A21 *	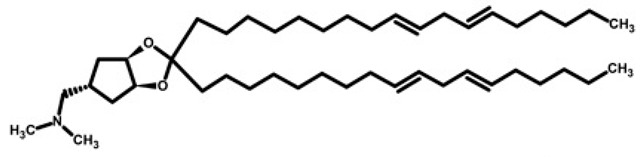	6.770	A41	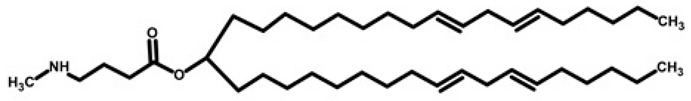	8.110
A22	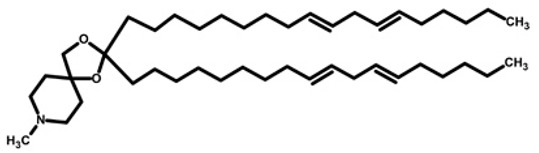	6.530	A42	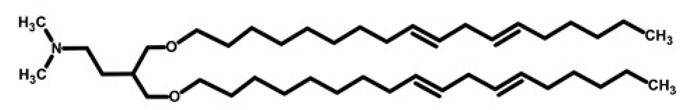	7.230
A23	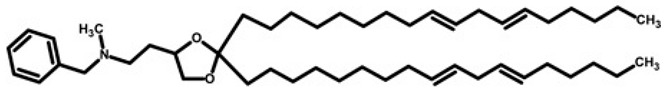	4.950	A43	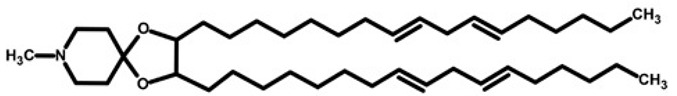	6.080
A24	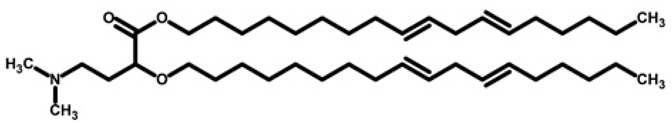	6.660	A44	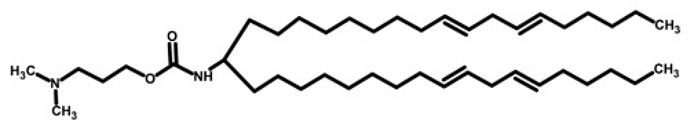	7.210
A25	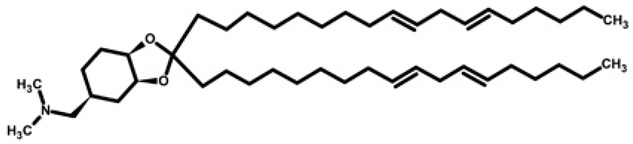	6.600	A45	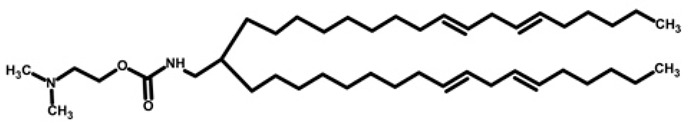	7.070
A26 *	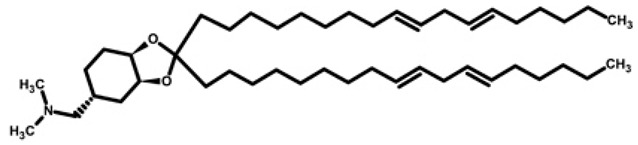	6.650	A46	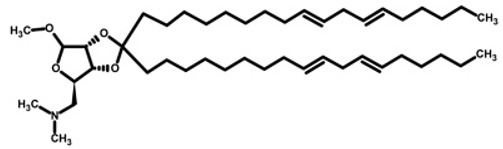	5.500
A27	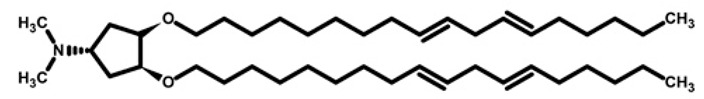	7.730	A47	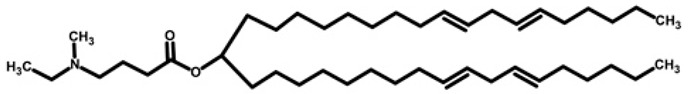	6.210
A28	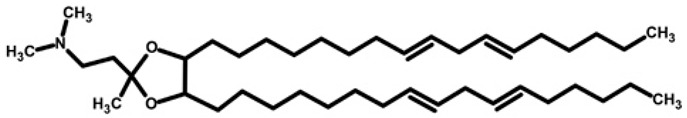	6.600	A48	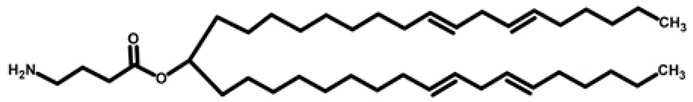	8.120
A29	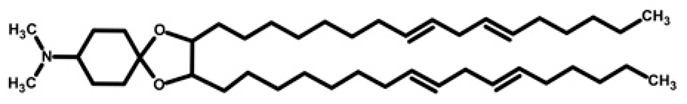	7.030	A49	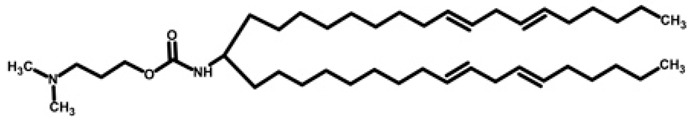	7.570
A30 *	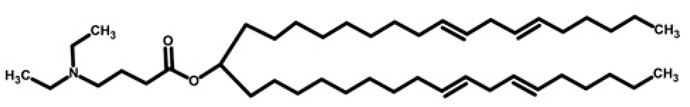	6.170	A50	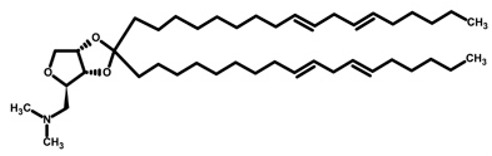	6.870
A31	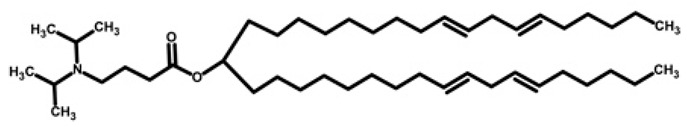	5.440	A51 *	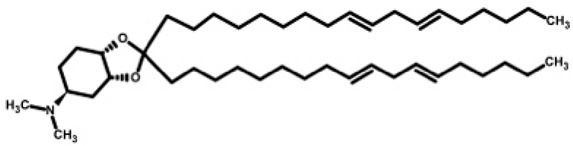	5.490
A32	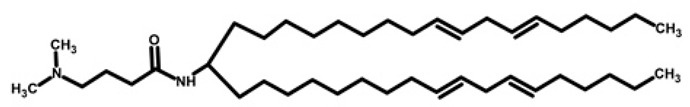	7.880	A52	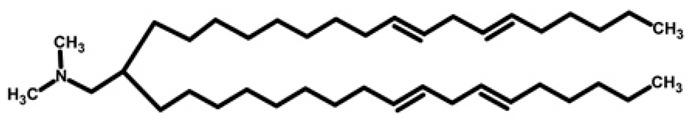	5.730
A33	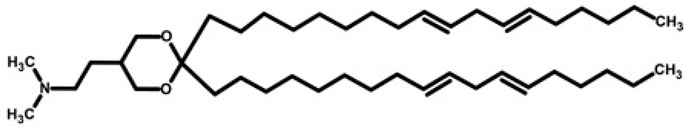	7.250	A53	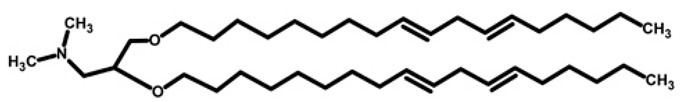	6.380
A34	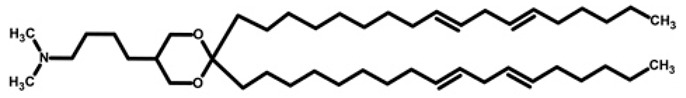	7.610	A54 *	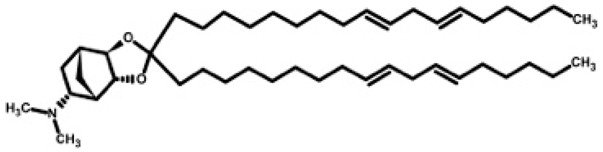	6.630
A35	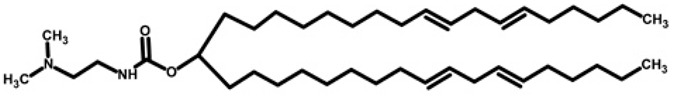	6.490	A55	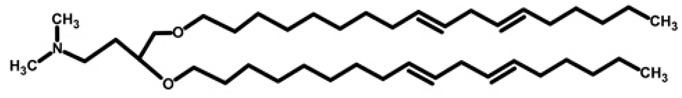	7.270
A36 *	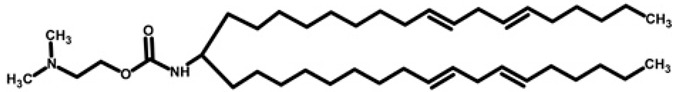	6.520	A56	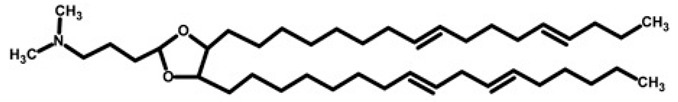	6.910

* Test set compounds.

## Data Availability

The original contributions presented in this study are included in the article/Supplementary Materials. Further inquiries can be directed to the corresponding author(s).

## Supplementary Materials

The following supporting information can be downloaded at: https://www.mdpi.com/article/10.3390/ijms27094075/s1.

## References

[1] Williams R., Aspinall R., Bellis M., Camps–Walsh G., Cramp M., Dhawan A., Ferguson J., Forton D., Foster G., Gilmore I. (2014). Addressing liver disease in the UK: A blueprint for attaining excellence in health care and reducing premature mortality from lifestyle issues of excess consumption of alcohol, obesity, and viral hepatitis. Lancet.

[2] Asrani S.K., Devarbhavi H., Eaton J., Kamath P.S. (2019). Burden of liver diseases in the world. J. Hepatol..

[3] Tan E.Y., Danpanichkul P., Yong J.N., Yu Z., Tan D.J.H., Lim W.H., Koh B., Lim R.Y.Z., Tham E.K.J., Mitra K. (2025). Liver cancer in 2021: Global Burden of Disease study. J. Hepatol..

[4] Li Q., Ding C., Cao M., Yang F., Yan X., He S., Cao M., Zhang S., Teng Y., Tan N. (2024). Global epidemiology of liver cancer 2022: An emphasis on geographic disparities. Chin. Med. J..

[5] Malakondaiah S., Julius A., Ponnambalam D., Gunthoti S., Ashok J., Krishana P., Rebecca J. (2024). Gene silencing by RNA interference: A review. Genome Instab. Dis..

[6] Dowdy S.F. (2017). Overcoming cellular barriers for RNA therapeutics. Nat. Biotechnol..

[7] Son S.Y., Geevarghese R., Valizadeh N., Yarmohammadi H. (2026). Advancing the frontiers of hepatocellular carcinoma treatment: A comprehensive review of past, ongoing, and future clinical trials. Chin. Clin. Oncol..

[8] Udepurkar A., Devos C., Sagmeister P., Destro F., Inguva P., Ahmadi S., Boulais E., Quan Y., Braatz R.D., Myerson A.S. (2025). Structure and Morphology of Lipid Nanoparticles for Nucleic Acid Drug Delivery: A Review. ACS Nano.

[9] Elnaggar M.G., He Y., Yeo Y. (2024). Recent trends in the delivery of RNA drugs: Beyond the liver, more than vaccine. Eur. J. Pharm. Biopharm..

[10] Ali Zaidi S.S., Fatima F., Ali Zaidi S.A., Zhou D., Deng W., Liu S. (2023). Engineering siRNA therapeutics: Challenges and strategies. J. Nanobiotechnol..

[11] Xu S., Hu Z., Song F., Xu Y., Han X. (2025). Lipid nanoparticles: Composition, formulation, and application. Mol. Ther. Methods Clin. Dev..

[12] Patel P., Ibrahim N.M., Cheng K. (2021). The Importance of Apparent pK_a_ in the Development of Nanoparticles Encapsulating siRNA and mRNA. Trends Pharmacol. Sci..

[13] Yan J., Zhang H., Li G., Su J., Wei Y., Xu C. (2024). Lipid nanovehicles overcome barriers to systemic RNA delivery: Lipid components, fabrication methods, and rational design. Acta Pharm. Sin. B.

[14] Reijenga J., van Hoof A., van Loon A., Teunissen B. (2013). Development of Methods for the Determination of pK_a_ Values. Anal. Chem. Insights.

[15] Dhumal D.M., Patil P.D., Kulkarni R.V., Akamanchi K.G. (2020). Experimentally Validated QSAR Model for Surface pK_a_ Prediction of Heterolipids Having Potential as Delivery Materials for Nucleic Acid Therapeutics. ACS Omega.

[16] Carrasco M.J., Alishetty S., Alameh M.G., Said H., Wright L., Paige M., Soliman O., Weissman D., Cleveland T.E., Grishaev A. (2021). Ionization and structural properties of mRNA lipid nanoparticles influence expression in intramuscular and intravascular administration. Commun. Biol..

[17] Li X., Li J., Wei J., Du W., Su C., Shen X., Zhao A., Xu M. (2025). Design Strategies for Novel Lipid Nanoparticle for mRNA Vaccine and Therapeutics: Current Understandings and Future Perspectives. MedComm.

[18] Wang W., Chen K., Jiang T., Wu Y., Wu Z., Ying H., Yu H., Lu J., Lin J., Ouyang D. (2024). Artificial intelligence–driven rational design of ionizable lipids for mRNA delivery. Nat. Commun..

[19] Zuchniarz J., Liu Y., Li C., Voth A.G. (2022). Accurate pK_a_ calculations in proteins with reactive molecular dynamics provide physical insight into the electrostatic origins of their values. J. Phys. Chem. B.

[20] Su K., Qiu J., Xu T., Liu S. (2026). Artificial intelligence–guided design of lipid nanoparticles for mRNA delivery. Acta Pharm. Sin. B.

[21] Li B., Raji I.O., Gordon A.G.R., Sun L., Raimondo T.M., Oladimeji F.A., Jiang A.Y., Varley A., Langer R.S., Anderson D.G. (2024). Accelerating ionizable lipid discovery for mRNA delivery using machine learning and combinatorial chemistry. Nat. Mater..

[22] Bhujel R., Enkmann V., Burgstaller H., Maharjan R. (2025). Artificial intelligence–driven strategies for targeted delivery and enhanced stability of RNA–based lipid nanoparticle cancer vaccines. Pharmaceutics.

[23] Kumar G., Ardekani A.M. (2025). Machine–Learning Framework to Predict the Performance of Lipid Nanoparticles for Nucleic Acid Delivery. ACS Appl. Bio Mater..

[24] DeCorte J., Brown B., Jeffrey R., Meiler J. (2025). Interpretable Deep–Learning pK_a_ Prediction for Small Molecule Drugs via Atomic Sensitivity Analysis. J. Chem. Inf. Model..

[25] Mayr F., Wieder M., Wieder O., Langer T. (2022). Improving Small Molecule pK_a_ Prediction Using Transfer Learning with Graph Neural Networks. Front. Chem..

[26] Gokcan H., Isayev O. (2022). Prediction of protein pK_a_ with representation learning. Chem. Sci..

[27] Dorsey P.J., Lau C.L., Chang T.C., Doerschuk P.C., D’Addio S.M. (2024). Review of machine learning for lipid nanoparticle formulation and process development. J. Pharm. Sci..

[28] Hanna A., Issadore D., Mitchell M. (2026). High–throughput platforms for machine learning–guided lipid nanoparticle design. Nat. Rev. Mater..

[29] Witten J., Raji I., Manan R.S., Beyer E., Bartlett S., Tang Y., Ebadi M., Lei J., Nguyen D., Oladimeji F. (2025). Artificial intelligence–guided design of lipid nanoparticles for pulmonary gene therapy. Nat. Biotechnol..

[30] Toopradab B., Xie W., Duan L., Hengphasatporn K., Harada R., Sinsulpsiri S., Shigeta Y., Shi L., Maitarad P., Rungrotmongkol T. (2024). Machine learning–based QSAR and LB–PaCS–MD guided design of SARS–CoV–2 main protease inhibitors. Bioorg. Med. Chem. Lett..

[31] Li H., Zhao Y., Xu C. (2025). Machine learning techniques for lipid nanoparticle formulation. Nano Converg..

[32] Xu Y., Ma S., Cui H., Chen J., Xu S., Gong F., Golubovic A., Zhou M., Wang C.W., Varley A. (2024). AGILE platform: A deep learning powered approach to accelerate LNP development for mRNA delivery. Nat. Commun..

[33] Xu Y., Cui H., Pang K., Li G., Gong F., Dong S., Wang B., Li B. (2026). LUMI–lab: A foundation model–driven autonomous platform enabling discovery of ionizable lipid designs for mRNA delivery. Cell.

[34] Xu Y., Gong F., Golubovic A., Strilchuk A., Chen J., Zhou M., Dong S., Seto B., Li B. (2025). Rational design and modular synthesis of biodegradable ionizable lipids via the Passerini reaction for mRNA delivery. Proc. Natl. Acad. Sci. USA.

[35] Jayaraman M., Ansell S.M., Mui B.L., Tam Y.K., Chen J., Du X., Butler D., Eltepu L., Matsuda S., Narayanannair J.K. (2012). Maximizing the potency of siRNA lipid nanoparticles for hepatic gene silencing in vivo. Angew. Chem. Int. Ed. Engl..

[36] Sato Y., Hashiba K., Sasaki K., Maeki M., Tokeshi M., Harashima H. (2019). Understanding structure–activity relationships of pH–sensitive cationic lipids facilitates the rational identification of promising lipid nanoparticles for delivering siRNAs in vivo. J. Control. Release.

[37] Topliss J.G., Edwards R.P. (1979). Chance factors in studies of quantitative structure–activity relationships. J. Med. Chem..

[38] Mansouri K., Cariello N.F., Korotcov A., Tkachenko V., Grulke C.M., Sprankle C.S., Allen D., Casey W.M., Kleinstreuer N.C., Williams A.J. (2019). Open–source QSAR models for pK_a_ prediction using multiple machine learning approaches. J. Cheminform..

[39] Abarbanel O.D., Hutchison G.R. (2024). QupKake: Integrating Machine Learning and Quantum Chemistry for Micro–pK_a_ Predictions. J. Chem. Theory Comput..

[40] (2014). Materials Studio Modeling.

[41] Bento A.P., Hersey A., Félix E., Landrum G., Gaulton A., Atkinson F., Bellis L.J., De Veij M., Leach A.R. (2020). An open source chemical structure curation pipeline using RDKit. J. Cheminform..

[42] Saptoro A., Tadé M. (2012). A Modified Kennard–Stone Algorithm for Optimal Division of Data for Developing Artificial Neural Network Models. Chem. Prod. Process Model..

[43] Daoud J. (2017). Multicollinearity and Regression Analysis. J. Phys. Conf. Ser..

[44] Schober P., Boer C., Schwarte L.A. (2018). Correlation Coefficients: Appropriate Use and Interpretation. Anesth. Analg..

[45] Salmerón R., Pérez J., Garcia C., López Martín M. (2017). A note about the corrected VIF. Stat. Pap..

[46] Lin D., Foster D., Ungar L. (2011). VIF Regression: A Fast Regression Algorithm for Large Data. J. Am. Stat. Assoc..

[47] García–Torres M., Saucedo F., Divina F., Gómez–Guerrero S. (2026). RFMSU: A multivariate symmetrical uncertainty–based random forest. Pattern Recognit..

[48] Cava W., Bauer C., Moore J.H., Pendergrass S.A. (2019). Interpretation of machine learning predictions for patient outcomes in electronic health records. AMIA Annu. Symp. Proc..

[49] Hu J., Szymczak S. (2023). A review on longitudinal data analysis with random forest. Brief. Bioinform..

[50] Zhou H., Wekesa J.S., Luan Y., Meng J. (2021). PRPI–SC: An ensemble deep learning model for predicting plant lncRNA–protein interactions. BMC Bioinform..

[51] Sagi O., Rokach L. (2021). Approximating XGBoost with an interpretable decision tree. Inf. Sci..

[52] Hernandez H. (2023). Replacing the R^2^ Coefficient in Model Analysis. Forsch. Res. Rep..

[53] Xie W., Wiriyarattanakul S., Rungrotmongkol T., Shi L., Wiriyarattanakul A., Maitarad P. (2023). Rational Design of a Low–Data Regime of Pyrrole Antioxidants for Radical Scavenging Activities Using Quantum Chemical Descriptors and QSAR with the GA–MLR and ANN Concepts. Molecules.

[54] Kiralj R., Ferreira M. (2009). Basic validation procedures for regression models in QSAR and QSPR studies: Theory and application. J. Braz. Chem. Soc..

[55] Abubakar Suleiman A., Abdullahi U.A., Ahmad A. (2015). An analysis of residuals in multiple regressions. Int. J. Adv. Sci. Eng..

[56] Verma V. (2025). A Comprehensive Framework for Residual Analysis in Regression and Machine Learning. J. Inf. Syst. Eng. Manag..

