# Supplementary material for: Machine Learning–Driven QSAR Modeling for pKa Prediction of Ionizable Lipids in Lipid Nanoparticles for Hepatic Gene Silencing
Source: Int J Mol Sci. 2026 May 1;27(9):4075. doi: 10.3390/ijms27094075 (PMC13164459; doi:10.3390/ijms27094075)
Supplement: Supplementary file 1 [file ijms-27-04075-s001.zip › ijms-4247424-supplementary.pdf]

Supporting Information

Machine Learning–Driven QSAR Modeling for pK_a Prediction of Ionizable Lipids in Lipid Nanoparticles for Hepatic Gene Silencing

Napat Kongtaworn [1,†], Borwornlak Toopradab [1,2,†], Duangjai Todsaporn [2], Poomrapee Tinpovong [1], Rada Thongsuebsaeng [2], Phornphimon Maitarad [2,3,*] and Thanyada Rungrotmongkol [1,2,*]

[1] Program in Bioinformatics and Computational Biology, College of Interdisciplinary and Integrative Studies, Chulalongkorn University, Bangkok 10330, Thailand; napat9079@gmail.com (N.K.); piyajaree@gmail.com (B.T.); poomrapee.tin@gmail.com (P.T.)
[2] Center of Excellence in Structural and Computational Biology, Department of Biochemistry, Faculty of Science, Chulalongkorn University, Bangkok 10330, Thailand; fai.dt8@gmail.com (D.T.); rada.mnm27@gmail.com (R.T.)
[3] Research Center of Nano Science and Technology, College of Science, Shanghai University, Shanghai 200444, China
* Correspondence: pmaitarad@shu.edu.cn or maitarad@hotmail.com (P.M.); thanyada.r@chula.ac.th or t.rungrotmongkol@gmail.com (T.R.)
† These authors contributed equally to this work.

List of supporting information

No.	Figure/Table	Page
1	**Table S1.** 47 molecular descriptors of 56 DLin-KC2 DMA derivatives.	**1**
2	**Figure S1.** Pearson correlation coefficients of pK_a to with related properties.	**8**
3	**Statistical Metrics Section**	**9**
3	**Table S2.** Hyperparameters to be tested for RF, ANN, and XGB.	**10**
4	**Table S3.** Experimental and predicted pK_a of 56 amino lipids of three models using GFA descriptor selection method.	**10**
5	**Table S4.** Experimental and predicted pK_a of 56 amino lipids of three models using VIF descriptor selection method.	**12**
6	**Table S5.** Experimental and predicted pK_a of 56 amino lipids of three models using PI descriptor selection method.	**14**
**7**	**Table S6. Calculated molecular descriptors of external validation set and their predicted pK_a and RMSE value from XGB models.**	**16**
8	**Code section**	**16**

Table S1. 47 molecular descriptors of 56 DLin-KC2 DMA derivatives.

Cpd.	X1	X2	X3	X4	X5	X6	X7	X8	X9	X10	X11	X12	X13	X14	X15	X16
A01*	0.362	0.705	0.454	-0.618	-0.846	-0.851	-0.988	-0.852	0.293	0.882	0.957	0.927	0.424	-0.843	1.360	-0.038
A02	0.408	2.471	-0.447	-0.660	0.329	-0.249	-0.118	-2.581	0.237	0.517	-0.354	-0.194	2.401	-0.016	1.473	-0.280
A03	-0.477	-1.245	0.186	0.371	0.349	0.539	1.004	1.401	-0.365	-1.306	-0.285	-0.638	-1.308	0.276	-0.790	0.336
A04	-0.690	-0.320	-0.179	-0.729	-0.294	1.113	0.312	0.747	0.746	-0.963	0.342	0.213	-0.291	-0.527	-1.386	-0.701
A05	0.144	-1.664	-1.357	-0.633	0.039	0.801	-0.861	1.708	0.591	0.726	-0.196	0.839	-1.080	-0.653	-2.182	-0.468
A06*	1.216	0.944	-1.253	0.319	-0.251	-0.798	-1.039	-1.517	-0.480	1.046	0.165	0.887	1.440	0.359	0.796	0.443
A07	-0.168	0.443	0.047	1.505	0.094	0.203	0.421	-1.009	-2.101	-0.471	-0.441	-0.325	0.975	1.468	0.488	1.981
A08*	0.467	0.328	-0.965	0.491	-0.197	0.051	-0.530	-0.174	-0.492	0.246	-0.349	0.666	0.140	0.717	0.150	0.324
A09	0.399	0.436	-0.780	1.028	0.538	0.011	-0.240	-0.435	-0.892	0.994	-0.615	-0.351	0.574	1.374	-0.154	0.560
A10	1.497	0.480	-2.002	-0.691	-0.007	-0.643	-1.525	-0.423	0.571	1.554	0.399	1.288	0.367	-0.482	0.305	-0.507
A11*	0.381	-0.406	-0.539	0.195	-0.369	-0.142	-0.323	0.923	-0.491	0.210	0.605	0.361	-0.898	0.753	-0.432	0.310
A12	0.693	0.132	-1.065	0.529	-0.264	-0.195	-0.808	-0.644	-0.678	1.160	0.010	0.434	0.639	0.416	0.273	0.661
A13*	-0.618	0.088	-0.071	0.153	-0.042	0.930	0.851	0.017	0.143	-1.102	-0.675	-0.545	-0.060	0.007	0.094	-0.175
A14	0.903	2.144	-0.457	-0.030	0.334	-0.943	-0.538	-2.017	0.556	0.157	-0.033	0.744	2.015	-0.670	0.823	-0.418
A15	1.004	1.424	-0.382	0.024	0.731	-1.142	-0.635	-1.549	0.721	0.260	-0.551	0.823	1.588	-0.679	0.535	-0.615
A16	0.989	0.811	0.200	1.146	0.856	-1.552	-0.824	-0.802	-0.664	-0.062	-1.038	1.364	0.799	0.596	0.331	0.577
A17	-0.543	-0.581	0.679	-2.823	-2.436	0.268	-0.299	0.901	2.232	0.463	3.059	0.136	-1.525	-0.723	1.118	-2.418
A18	-0.785	-1.018	-0.537	-0.923	-1.864	1.513	0.833	-0.074	0.423	-0.286	1.158	-1.120	0.709	1.365	-1.478	-1.020
A19	-0.655	-1.171	0.077	-0.980	0.027	0.872	0.500	0.499	1.232	-0.044	0.009	-0.767	-0.498	-0.149	-0.203	-1.428
A20*	-1.014	-0.124	0.638	0.911	-0.117	0.967	1.715	0.140	-1.280	-1.450	-0.124	-1.668	0.003	0.863	-0.397	1.218
A21*	-0.561	-0.183	0.166	0.808	-0.128	0.674	1.206	0.078	-0.999	-0.830	-0.008	-1.314	0.098	0.717	-0.448	0.935
A22	-0.657	-0.389	0.779	0.143	-0.198	0.356	1.157	-0.046	-0.207	-1.342	0.744	-0.856	0.293	-0.110	-0.569	0.290
A23	-2.272	-0.448	3.140	0.759	1.726	0.900	1.863	2.894	-0.181	-2.189	-2.000	-1.359	-3.417	-2.899	0.070	1.302
A24	-2.310	1.800	2.057	-0.670	-0.239	1.757	1.635	-1.419	0.343	-1.472	1.535	-1.525	1.212	0.256	1.068	-0.509
A25	-1.256	-0.339	0.810	1.144	-0.462	1.183	1.439	0.388	-1.584	-1.679	-0.301	-1.057	-0.395	1.607	-0.142	1.306
A26*	0.079	-0.872	-0.438	1.034	-0.128	0.212	-0.312	1.099	-1.309	0.210	-0.910	0.343	-1.058	1.456	-0.543	1.031
A27	-0.709	-0.698	0.142	0.149	-0.136	0.902	0.461	0.877	0.112	-0.201	0.133	-0.589	-0.819	0.291	-0.491	-0.244
A28	0.457	-0.116	-0.385	-0.596	0.061	-0.364	-0.855	0.251	0.565	0.226	0.461	1.200	-0.299	-0.884	0.012	-0.350

Table S1. 47 molecular descriptors of 56 DLin-KC2 DMA derivatives. (cont.)

Cpd.	X1	X2	X3	X4	X5	X6	X7	X8	X9	X10	X11	X12	X13	X14	X15	X16
A29	-0.105	0.715	-0.480	0.401	-1.313	0.505	0.539	-1.099	-0.410	-0.499	1.110	-0.493	1.087	0.280	0.474	0.390
A30*	-0.061	-0.622	0.577	-1.586	-2.254	-0.341	0.147	0.056	0.759	-0.469	1.669	0.112	0.062	0.551	-0.301	-1.121
A31	-0.245	0.344	0.573	0.343	1.546	-0.076	0.413	-0.308	0.324	-0.583	-0.675	-0.230	0.100	-0.796	0.620	-0.092
A32	0.387	0.755	1.340	0.885	1.681	-1.542	-0.074	-0.871	0.081	-0.782	-1.434	0.697	0.175	-0.253	2.007	-0.003
A33	-0.456	0.719	0.157	0.419	0.015	0.531	0.954	-0.266	-1.176	-1.171	-0.070	-0.658	0.245	0.671	0.158	1.165
A34	-0.403	-1.456	0.081	-0.437	0.014	0.512	0.639	1.139	0.543	-0.459	0.029	-0.682	-1.175	-0.514	-0.377	-0.462
A35	0.362	-1.894	-0.713	-0.154	1.510	0.015	-0.472	0.683	0.805	1.139	-0.775	-0.088	-0.567	-1.398	-0.550	-0.446
A36*	0.473	-0.782	1.294	0.867	1.773	-1.630	-0.564	0.135	-0.259	0.116	-1.651	0.816	-0.432	1.376	0.651	-0.203
A37	0.599	0.107	-0.034	0.417	1.003	-0.825	-0.211	0.428	-0.018	0.417	-0.793	0.029	-0.386	-0.525	-0.274	0.218
A38	0.957	0.176	-0.211	-1.999	-1.546	-1.202	-0.956	-0.375	0.965	0.652	1.225	1.046	0.692	0.127	-0.602	-1.210
A39	-0.411	-0.298	0.283	0.005	-0.096	0.374	0.151	0.113	-0.578	0.296	-0.122	-0.461	0.285	0.476	-0.992	0.518
A40	2.070	1.661	-0.821	0.540	0.221	-2.330	-1.436	-1.686	0.249	0.758	0.005	1.735	1.862	0.282	0.264	-0.405
A41	-0.201	0.452	0.297	-0.857	-1.176	0.065	0.389	-0.661	0.205	-0.521	0.848	-0.237	0.653	-0.138	0.286	-0.195
A42	-0.474	-0.894	0.012	0.720	-0.997	0.664	-0.099	0.407	-1.069	0.674	0.931	-0.340	-0.395	0.897	-0.193	0.951
A43	-0.796	0.521	0.824	-0.638	0.594	0.520	-0.126	-0.059	1.206	0.022	0.700	0.185	0.499	-0.777	-1.021	-1.162
A44	0.778	0.277	1.118	0.618	2.574	-1.933	-1.013	0.133	-0.095	0.434	-1.939	1.299	-1.270	-1.713	2.647	0.755
A45	1.475	1.376	0.084	1.655	2.517	-2.156	-2.237	-1.071	-1.398	1.123	-2.945	2.746	0.152	-1.310	2.615	2.174
A46	-0.599	0.063	-0.008	-2.623	-0.811	0.856	0.957	-0.150	2.670	-0.427	1.021	-1.215	0.392	-1.975	-0.513	-2.477
A47	1.946	0.441	-1.688	1.525	-0.003	-1.513	-1.789	0.261	-0.825	1.618	0.030	1.663	-0.082	1.144	-0.529	0.565
A48	2.234	-0.797	-2.736	-0.508	-0.227	-1.145	-1.969	0.840	1.392	3.034	0.247	0.903	-0.837	-1.441	-0.345	-1.138
A49	0.401	-2.530	-0.100	0.321	0.009	-0.495	0.201	1.836	-1.001	0.051	0.279	-0.359	-1.942	1.396	-0.491	0.683
A50	-0.900	-1.335	0.179	-2.508	-0.934	1.144	1.334	1.358	2.826	-0.775	1.334	-1.559	-0.943	-2.048	-1.537	-2.637
A51	1.074	-0.417	-1.298	0.720	-0.510	-0.564	-1.245	0.394	-0.829	1.161	-0.203	1.131	-0.423	1.083	-0.090	0.593
A52	-1.146	-0.638	0.844	-0.180	-0.342	1.002	0.796	0.375	0.135	-0.341	0.195	-1.021	0.336	-0.376	-1.841	-0.022
A53	-1.914	-0.102	1.613	-0.855	-0.012	1.523	1.931	0.546	0.936	-2.308	-0.020	-1.380	-0.368	-0.608	-0.642	-0.900
A54*	0.894	0.016	-1.218	0.444	-0.541	-0.367	-0.957	-0.164	-0.663	0.994	-0.252	0.795	0.035	0.953	0.372	0.442
A55	-1.475	1.709	1.502	0.142	-0.317	0.982	1.047	-0.833	-0.628	-0.021	0.817	-1.658	0.136	0.729	1.992	0.483
A56	-0.749	-0.200	0.011	0.967	0.517	1.056	0.151	0.460	-1.195	0.612	-1.255	-0.695	0.080	0.021	-1.469	1.431

Table S1. 47 molecular descriptors of 56 DLin-KC2 DMA derivatives. (cont.)

Cpd.	X17	X18	X19	X20	X21	X22	X23	X24	X25	X26	X27	X28	X29	X30	X31	X32
A01*	-0.689	-0.540	-0.495	-0.426	-0.362	-0.452	-0.409	-0.354	0.195	-0.304	-0.280	-0.428	-0.356	-0.393	0.488	0.488
A02	-0.743	-0.541	-0.495	-0.426	-0.362	-0.452	-0.409	-0.354	-0.073	-0.305	-0.280	-0.429	-0.356	-0.393	0.488	0.488
A03	-0.787	-0.570	-1.014	-0.640	-0.483	-1.013	-0.631	-0.478	0.479	-0.722	-0.707	-0.970	-0.842	-0.961	0.488	0.488
A04	-0.689	-0.540	-0.495	-0.426	-0.362	-0.452	-0.409	-0.354	0.195	-0.304	-0.280	-0.428	-0.356	-0.393	0.488	0.488
A05	-0.689	-0.540	-0.495	-0.426	-0.362	-0.452	-0.409	-0.354	0.195	-0.304	-0.280	-0.428	-0.356	-0.393	0.488	0.488
A06*	-0.785	-1.083	-0.940	-1.277	-1.258	-0.931	-1.282	-1.259	0.804	0.554	0.579	0.121	-0.097	0.093	0.488	0.488
A07	-0.785	-1.083	-0.940	-1.277	-1.258	-0.931	-1.282	-1.259	0.804	0.554	0.579	0.121	-0.097	0.093	0.488	0.488
A08*	0.125	-1.051	-0.422	-1.074	-1.153	-0.372	-1.071	-1.151	0.670	1.003	1.011	0.689	0.389	0.661	0.488	0.488
A09	0.125	-1.051	-0.422	-1.074	-1.153	-0.372	-1.071	-1.151	0.670	1.003	1.011	0.689	0.389	0.661	0.488	0.488
A10	-0.591	-0.487	0.024	-0.211	-0.244	0.109	-0.186	-0.233	-0.101	0.121	0.149	0.125	0.129	0.175	0.488	0.488
A11*	-1.026	-1.134	-1.975	-1.444	-1.282	-2.049	-1.458	-1.286	0.543	-0.312	-0.280	-0.974	-1.070	-1.043	1.927	1.927
A12	-0.928	-1.102	-1.458	-1.238	-1.175	-1.490	-1.245	-1.176	0.405	0.115	0.149	-0.435	-0.583	-0.475	1.927	1.927
A13*	-0.831	-1.079	-0.940	-1.032	-1.070	-0.931	-1.031	-1.069	0.071	0.553	0.579	0.119	-0.097	0.093	1.927	1.927
A14	-0.018	1.015	-0.506	0.464	0.798	-0.627	0.441	0.790	0.221	-1.511	-1.556	-1.487	-1.329	-1.529	-0.951	-0.951
A15	0.372	1.073	0.013	0.687	0.926	-0.066	0.673	0.921	0.400	-1.116	-1.132	-0.960	-0.842	-0.961	-0.951	-0.951
A16	0.876	1.123	0.533	0.909	1.059	0.495	0.904	1.059	0.089	-0.710	-0.707	-0.419	-0.356	-0.393	-0.951	-0.951
A17	1.593	1.165	1.052	1.132	1.187	1.056	1.136	1.191	-0.215	-0.292	-0.280	0.137	0.129	0.175	-0.951	-0.951
A18	2.309	1.200	1.571	1.355	1.321	1.618	1.368	1.329	-0.513	0.137	0.149	0.708	0.614	0.742	-0.951	-0.951
A19	0.320	-0.462	0.543	0.003	-0.123	0.670	0.037	-0.108	-0.390	0.562	0.579	0.696	0.614	0.742	0.488	0.488
A20*	0.122	-1.066	-0.422	-1.074	-1.060	-0.372	-1.071	-1.057	1.135	1.005	1.011	0.691	0.389	0.661	0.488	0.488
A21*	0.122	-1.066	-0.422	-1.074	-1.060	-0.372	-1.071	-1.057	1.135	1.005	1.011	0.691	0.389	0.661	0.488	0.488
A22	-1.101	-1.077	-0.940	-1.277	-1.166	-0.931	-1.282	-1.166	-0.351	0.553	0.579	0.119	-0.097	0.093	1.927	1.927
A23	-0.009	-1.022	1.649	0.006	-0.418	1.390	-0.137	-0.499	1.549	2.930	2.754	3.123	2.368	2.727	0.488	0.488
A24	-0.866	1.000	1.571	0.986	0.699	1.410	0.901	0.644	-0.086	0.105	0.149	0.669	1.247	0.821	-0.951	-0.951
A25	0.839	-1.033	0.095	-0.869	-0.955	0.188	-0.859	-0.950	1.047	1.466	1.445	1.274	0.874	1.229	0.488	0.488
A26*	0.839	-1.033	0.095	-0.869	-0.955	0.188	-0.859	-0.950	1.047	1.466	1.445	1.274	0.874	1.229	0.488	0.488
A27	-1.400	-0.683	-0.495	-0.122	-0.362	-0.452	-0.095	-0.354	-1.669	-0.320	-0.280	-0.450	-0.356	-0.393	-0.951	-0.951
A28	-1.022	-0.509	-1.014	-0.923	-0.616	-1.013	-0.923	-0.613	1.393	-0.719	-0.707	-0.964	-0.842	-0.961	0.488	0.488

Table S1. 47 molecular descriptors of 56 DLin-KC2 DMA derivatives. (cont.)

Cpd.	X17	X18	X19	X20	X21	X22	X23	X24	X25	X26	X27	X28	X29	X30	X31	X32
A29	-0.397	-1.045	-0.940	-1.277	-1.258	-0.931	-1.282	-1.259	0.901	0.558	0.579	0.126	-0.097	0.093	0.488	0.488
A30*	1.972	1.231	1.571	1.355	0.894	1.618	1.368	0.892	-0.072	0.135	0.149	0.707	0.614	0.742	-0.951	-0.951
A31	3.158	1.353	2.610	1.078	0.769	2.740	1.085	0.766	0.832	1.023	1.011	1.889	1.583	1.878	-0.951	-0.951
A32	-0.140	1.134	0.533	0.909	1.059	0.405	0.867	1.038	0.089	-0.710	-0.707	-0.419	-0.245	-0.433	-0.951	-0.951
A33	-0.390	-0.516	0.024	-0.211	-0.244	0.109	-0.186	-0.233	-0.397	0.124	0.149	0.127	0.129	0.175	0.488	0.488
A34	1.042	-0.478	1.063	0.218	-0.005	1.231	0.261	0.013	-0.933	1.015	1.011	1.283	1.099	1.310	0.488	0.488
A35	0.184	1.111	0.533	0.909	1.059	0.383	0.858	1.032	0.089	-0.710	-0.707	-0.419	0.354	-0.353	-0.951	-0.951
A36*	0.184	1.112	0.533	0.909	1.059	0.383	0.858	1.032	0.089	-0.710	-0.707	-0.419	0.354	-0.353	-0.951	-0.951
A37	1.299	1.158	1.052	1.522	1.187	1.056	1.541	1.191	-1.725	-0.291	-0.280	0.138	0.129	0.175	-0.951	-0.951
A38	1.175	1.168	1.052	1.132	1.187	1.056	1.136	1.191	-0.215	-0.292	-0.280	0.137	0.129	0.175	-0.951	-0.951
A39	1.179	1.085	0.013	0.687	0.926	0.119	0.749	0.966	-0.965	-1.116	-1.132	-0.960	-1.455	-0.999	-0.951	-0.951
A40	-0.125	1.123	0.533	0.909	1.059	0.405	0.867	1.038	-0.035	-0.709	-0.707	-0.418	-0.245	-0.433	-0.951	-0.951
A41	0.035	1.066	0.013	1.075	0.926	-0.066	1.075	0.921	-1.145	-1.115	-1.132	-0.959	-0.842	-0.961	-0.951	-0.951
A42	-1.005	0.777	0.533	1.299	1.292	0.658	1.376	1.338	-2.900	-0.730	-0.707	-0.447	-0.134	-0.311	-0.951	-0.951
A43	-0.844	-1.092	-1.975	-1.444	-1.282	-2.049	-1.458	-1.286	0.646	-0.308	-0.280	-0.969	-1.070	-1.043	1.927	1.927
A44	0.282	1.154	1.052	1.132	1.187	0.944	1.090	1.164	-0.215	-0.292	-0.280	0.137	0.845	0.215	-0.951	-0.951
A45	0.703	1.146	1.052	1.132	1.187	0.944	1.090	1.164	-0.325	-0.291	-0.280	0.138	0.845	0.215	-0.951	-0.951
A46	-1.079	-0.998	0.613	-0.905	-1.211	0.702	-0.911	-1.215	1.827	1.933	1.880	1.870	2.242	1.957	1.927	1.927
A47	1.424	1.175	1.052	1.132	0.968	1.056	1.136	0.967	0.461	-0.293	-0.280	0.137	0.129	0.175	-0.951	-0.951
A48	-0.643	1.013	-0.506	0.851	0.798	-0.627	0.842	0.790	-0.845	-1.510	-1.556	-1.486	-1.329	-1.529	-0.951	-0.951
A49	0.801	1.181	1.571	1.355	1.321	1.505	1.322	1.302	-0.609	0.137	0.149	0.709	1.336	0.783	-0.951	-0.951
A50	-1.187	-1.057	-0.422	-1.074	-1.153	-0.394	-1.080	-1.156	1.046	1.003	1.011	0.689	0.865	0.741	1.927	1.927
A51	0.125	-1.051	-0.422	-1.074	-1.153	-0.372	-1.071	-1.151	0.670	1.003	1.011	0.689	0.389	0.661	0.488	0.488
A52	0.961	0.799	-2.065	0.180	0.632	-2.103	0.230	0.668	-1.455	-2.627	-2.815	-2.978	-4.072	-3.311	-0.951	-0.951
A53	-1.402	0.715	-0.506	0.851	1.029	-0.464	0.910	1.067	-2.349	-1.525	-1.556	-1.508	-1.105	-1.447	-0.951	-0.951
A54*	0.032	-1.331	-0.816	-1.753	-1.805	-0.793	-1.766	-1.805	1.154	1.929	1.880	1.269	0.645	1.147	0.488	0.488
A55	-1.304	0.787	0.013	1.075	1.158	0.097	1.143	1.199	-2.634	-1.130	-1.132	-0.980	-0.619	-0.879	-0.951	-0.951
A56	-0.725	-0.544	-1.014	-0.338	-0.341	-1.013	-0.320	-0.334	-0.630	-0.714	-0.707	-0.960	-0.842	-0.961	0.488	0.488

Table S1. 47 molecular descriptors of 56 DLin-KC2 DMA derivatives. (cont.)

Cpd.	X33	X34	X35	X36	X37	X38	X39	X40	X41	X42	X43	X44	X45	X46	X47
A01*	-0.564	-0.397	0.140	-0.152	0.538	0.118	-0.380	0.109	-0.266	0.118	-0.443	-0.244	-0.408	-0.244	0.227
A02	-0.564	-0.397	0.140	-0.419	0.538	0.118	-0.380	0.109	-0.266	0.118	-0.443	-0.244	-0.408	-0.244	0.227
A03	-0.564	-0.925	0.140	-0.846	0.538	0.118	-0.993	0.109	-0.266	0.118	-0.443	-0.244	-0.966	-0.244	0.227
A04	-0.564	-0.397	0.140	-0.152	0.538	0.118	-0.380	0.109	-0.266	0.118	-0.443	-0.244	-0.408	-0.244	0.227
A05	-0.564	-0.397	0.140	-0.152	0.538	0.118	-0.380	0.109	-0.266	0.118	-0.443	-0.244	-0.408	-0.244	0.227
A06*	-0.564	0.130	1.256	0.097	0.538	1.219	0.029	0.109	-0.266	1.219	-0.443	-0.244	0.149	-0.244	0.803
A07	-0.564	0.130	1.256	0.097	0.538	1.219	0.029	0.109	-0.266	1.219	-0.443	-0.244	0.149	-0.244	0.803
A08*	-0.564	0.658	1.256	0.790	0.538	1.219	0.642	0.109	-0.266	1.219	-0.443	-0.244	0.707	-0.244	0.803
A09	-0.564	0.658	1.256	0.790	0.538	1.219	0.642	0.109	-0.266	1.219	-0.443	-0.244	0.707	-0.244	0.803
A10	-0.564	0.130	0.140	0.541	0.538	0.118	0.234	0.109	-0.266	0.118	-0.443	-0.244	0.149	-0.244	0.803
A11*	-0.564	-0.925	1.256	-1.290	0.538	1.219	-1.197	0.109	-0.266	1.219	-0.443	-0.244	-0.966	-0.244	0.227
A12	-0.564	-0.397	0.140	-0.597	0.538	0.118	-0.584	0.109	-0.266	1.219	-0.443	-0.244	-0.408	-0.244	0.227
A13*	-0.564	0.130	1.256	0.097	0.538	1.219	0.029	0.109	-0.266	1.219	-0.443	-0.244	0.149	-0.244	0.803
A14	0.248	-1.453	-0.977	-1.201	-0.831	-0.983	-1.606	0.109	-0.266	-0.983	1.076	-0.244	-1.523	-0.244	-1.504
A15	0.248	-0.925	-0.977	-0.508	-0.831	-0.983	-0.993	0.109	-0.266	-0.983	0.974	-0.244	-0.966	-0.244	-0.927
A16	0.248	-0.397	-0.977	0.186	-0.831	-0.983	-0.380	0.109	-0.266	-0.983	0.974	-0.244	-0.408	-0.244	-0.927
A17	0.248	0.130	-0.977	0.879	-0.831	-0.983	0.234	0.109	0.609	-0.983	0.974	-0.244	0.149	-0.244	-0.350
A18	0.248	0.658	-0.977	1.573	-0.831	-0.983	0.847	0.109	1.484	-0.983	0.974	-0.244	0.707	-0.244	-0.350
A19	-0.564	0.658	0.140	1.235	0.538	0.118	0.847	0.109	-0.266	0.118	-0.443	-0.244	0.707	-0.244	0.803
A20*	-0.564	0.658	1.256	0.541	0.538	1.219	0.642	0.109	-0.266	1.219	-0.443	-0.244	0.707	-0.244	0.803
A21*	-0.564	0.658	1.256	0.541	0.538	1.219	0.642	0.109	-0.266	1.219	-0.443	-0.244	0.707	-0.244	0.803
A22	-0.564	0.130	1.256	0.097	0.538	1.219	0.029	0.109	-0.266	1.219	-0.443	-0.244	0.149	-0.244	0.803
A23	0.771	2.857	0.140	1.555	0.538	1.219	2.482	3.155	-0.266	0.118	-0.443	1.464	2.937	1.464	-4.387
A24	2.971	1.009	-0.977	-2.268	0.538	-0.983	0.642	0.109	1.484	-0.983	1.076	3.172	0.707	3.172	-2.080
A25	-0.564	1.186	1.256	1.235	0.538	1.219	1.255	0.109	-0.266	1.219	-0.443	-0.244	1.264	-0.244	1.380
A26*	-0.564	1.186	1.256	1.235	0.538	1.219	1.255	0.109	-0.266	1.219	-0.443	-0.244	1.264	-0.244	1.380
A27	-0.564	-0.397	0.140	-0.775	0.538	0.118	-0.380	0.109	1.484	0.118	-1.253	-0.244	-0.408	-0.244	0.227
A28	-0.564	-0.925	0.140	-0.846	0.538	0.118	-0.993	0.109	-2.016	0.118	-0.443	-0.244	-0.966	-0.244	0.227

Table S1. 47 molecular descriptors of 56 DLin-KC2 DMA derivatives. (cont.)

Cpd.	X33	X34	X35	X36	X37	X38	X39	X40	X41	X42	X43	X44	X45	X46	X47
**A29**	-0.564	0.130	1.256	0.097	0.538	1.219	0.029	0.109	-2.016	1.219	-0.443	-0.244	0.149	-0.244	0.803
**A30***	0.248	0.658	-0.977	1.573	-0.831	-0.983	0.847	0.109	-0.266	-0.983	0.974	-0.244	0.707	-0.244	-0.350
**A31**	0.248	1.714	-0.977	2.942	-0.831	-0.983	2.073	0.109	-0.266	-0.983	0.974	-0.244	1.822	-0.244	0.227
**A32**	0.538	-0.485	-0.977	-0.579	-2.200	-0.983	-0.380	0.109	-0.266	-0.983	0.063	-0.244	-0.408	-0.244	-0.927
**A33**	-0.564	0.130	0.140	0.275	0.538	0.118	0.234	0.109	-0.266	0.118	-0.443	-0.244	0.149	-0.244	0.803
**A34**	-0.564	1.186	0.140	1.662	0.538	0.118	1.460	0.109	0.609	0.118	-0.443	-0.244	1.264	-0.244	1.380
**A35**	1.494	-0.310	-0.977	-0.881	-0.831	-0.983	-0.380	0.109	-0.266	-0.983	1.987	1.464	-0.408	1.464	-0.927
**A36***	1.494	-0.310	-0.977	-0.881	-0.831	-0.983	-0.380	0.109	-0.266	-0.983	1.987	1.464	-0.408	1.464	-0.927
**A37**	1.158	0.130	-0.977	0.968	-0.831	-0.983	0.234	-2.937	2.359	-0.983	0.974	-0.244	0.149	-0.244	-0.350
**A38**	1.158	0.130	-0.977	0.951	-0.831	-0.983	0.234	-2.937	0.609	-0.983	0.974	-0.244	0.149	-0.244	-0.350
**A39**	-2.140	-1.188	-0.977	-0.081	-2.200	-0.983	-0.788	3.155	-0.266	-0.983	-2.063	-1.952	-0.966	-1.952	0.227
**A40**	0.538	-0.485	-0.977	-0.828	-2.200	-0.983	-0.380	0.109	-0.266	-0.983	0.063	-0.244	-0.408	-0.244	-0.927
**A41**	1.158	-0.925	-0.977	-0.419	-0.831	-0.983	-0.993	-2.937	0.609	-0.983	0.974	-0.244	-0.966	-0.244	-0.927
**A42**	-0.564	-0.397	-0.977	-0.597	0.538	-0.983	-0.175	0.109	3.234	-0.983	-1.658	-0.244	-0.408	-0.244	0.227
**A43**	-0.564	-0.925	1.256	-1.290	0.538	1.219	-1.197	0.109	-2.016	1.219	-0.443	-0.244	-0.966	-0.244	0.227
**A44**	1.494	0.218	-0.977	-0.188	-0.831	-0.983	0.234	0.109	0.609	-0.983	1.987	1.464	0.149	1.464	-0.927
**A45**	1.494	0.218	-0.977	-0.437	-0.831	-0.983	0.234	0.109	-0.266	-0.983	1.987	1.464	0.149	1.464	-0.927
**A46**	1.348	2.065	1.256	-1.308	3.275	1.219	1.869	0.109	-0.266	1.219	-0.240	3.172	1.822	3.172	0.803
**A47**	0.248	0.130	-0.977	0.879	-0.831	-0.983	0.234	0.109	-0.266	-0.983	0.974	-0.244	0.149	-0.244	-0.350
**A48**	2.607	-1.453	-0.977	-0.899	-0.831	-0.983	-1.606	-2.937	-0.266	-0.983	0.974	-0.244	-1.523	-0.244	-1.504
**A49**	1.494	0.746	-0.977	0.257	-0.831	-0.983	0.847	0.109	0.609	-0.983	1.987	1.464	0.707	1.464	-0.350
**A50**	0.392	0.834	1.256	-1.273	1.906	1.219	0.642	0.109	-0.266	1.219	-0.443	1.464	0.707	1.464	0.803
**A51**	-0.564	0.658	1.256	0.790	0.538	1.219	0.642	0.109	-0.266	1.219	-0.443	-0.244	0.707	-0.244	0.803
**A52**	-2.475	-3.387	-0.977	0.044	-2.200	-0.983	-3.241	0.109	-0.266	-0.983	-2.164	-3.660	-3.195	-3.660	-0.350
**A53**	-0.564	-1.453	-0.977	-1.717	0.538	-0.983	-1.401	0.109	2.359	-0.983	-1.253	-0.244	-1.523	-0.244	-0.350
**A54***	-0.564	1.186	2.372	0.524	0.538	2.320	1.051	0.109	-0.266	2.320	-0.443	-0.244	1.264	-0.244	1.380
**A55**	-0.564	-0.925	-0.977	-1.024	0.538	-0.983	-0.788	0.109	2.359	-0.983	-1.354	-0.244	-0.966	-0.244	0.227
**A56**	-0.564	-0.925	0.140	-0.846	0.538	0.118	-0.993	0.109	-2.016	0.118	-0.544	-0.244	-0.966	-0.244	0.227

The compounds that have mark with asterisk are test set.

Properties X1~X47: Quadrupole xx, Quadrupole xy, Quadrupole yy, Quadrupole xz, Quadrupole yz, Quadrupole zz, Octupole xxx, Octupole xxy, Octupole xxz, Octupole xyy, Octupole xyz, Octupole xzz, Octupole yyy, Octupole yyz, Octupole yzz, Octupole zzz, AlogP98, Balaban index JX, Kappa-1, Kappa-2, Kappa-3, Kappa-1 (alpha modified), Kappa-2 (alpha modified), Kappa-3 (alpha modified), Bond information content (BIC), Edge distance/magnitude, Vertex adjacency/magnitude, Vertex distance/magnitude, Atomic composition (total), ExactMolWt, NumSaturatedHeterocycles, NumAliphaticHeterocycles, TPSA, HeavyAtomMolWt, NumAliphaticRings, MolLogP, fr_ether, RingCount, NumValenceElectrons, fr_NH0, fr_unbrch_alkane, NumSaturatedRings, MinAbsPartialCharge, NumHeteroatoms, HeavyAtomCount, NOCount, FractionCSP3 (calculated molecular descriptors by Material Studio and RDKit).

X1	Quadrupole xx	X17	AlogP98	X33	TPSA
X2	Quadrupole xy	X18	Balaban index JX	X34	HeavyAtomMolWt
X3	Quadrupole yy	X19	Kappa-1	X35	NumAliphaticRings
X4	Quadrupole xz	X20	Kappa-2	X36	MolLogP
X5	Quadrupole yz	X21	Kappa-3	X37	fr_ether
X6	Quadrupole zz	X22	Kappa-1 (alpha modified)	X38	RingCount
X7	Octupole xxx	X23	Kappa-2 (alpha modified)	X39	NumValenceElectrons
X8	Octupole xxy	X24	Kappa-3 (alpha modified)	X40	fr_NH0
X9	Octupole xxz	X25	Bond information content (BIC)	X41	fr_unbrch_alkane
X10	Octupole xyy	X26	Edge distance/magnitude	X42	NumSaturatedRings
X11	Octupole xyz	X27	Vertex adjacency/magnitude	X43	MinAbsPartialCharge
X12	Octupole xzz	X28	Atomic composition (total)	X44	NumHeteroatoms
X13	Octupole yyy	X29	Vertex distance/magnitude	X45	HeavyAtomCount
X14	Octupole yyz	X30	ExactMolWt	X46	NOCount
X15	Octupole yzz	X31	NumSaturatedHeterocycles	X47	FractionCSP3
X16	Octupole zzz	X32	NumAliphaticHeterocycles		

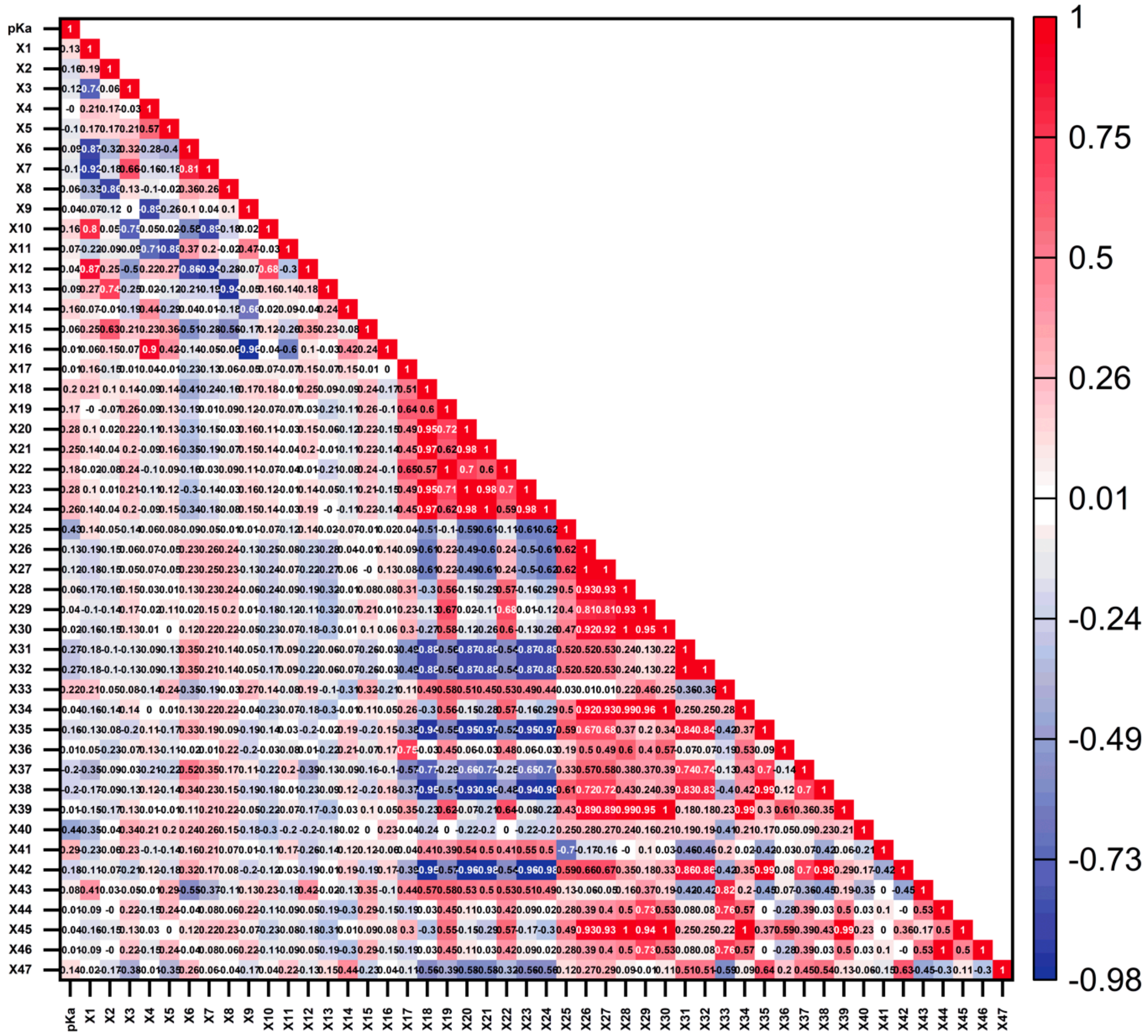

Figure S1. Pearson correlation coefficients of pK_a to with related properties.

Statistical Metrics Section

To rigorously evaluate the predictive performance of the developed ML-QSAR models, the entire dataset comprising fifty-six amino lipids was partitioned into training and test subsets using the Kennard-Stone algorithm. This algorithm systematically selects compounds that span the chemical space of the dataset, thereby maximizing structural diversity and ensuring that both subsets are representative of the overall chemical domain. The training set was subsequently used to construct the QSAR models during the model development stage, while the remaining compounds—unseen during training—served as the test set to evaluate external predictive capability.

The performance of all models was assessed using the coefficient of determination (R^2), which indicates the proportion of the total variance in the dependent variable explained by the model. An R^2 value closer to 1 signifies a better fit. Additionally, the root mean square error (RMSE) for both the training and test sets was calculated to validate the models. RMSE provides an estimate of the model's accuracy and its ability to generalize unseen data. R^2 and RMSE were calculated as in Equations (2) and (3), respectively. The cross-validated R^2, denoted as R^2cv, is the cross-validation equivalent of R^2 and serves as an essential measure of a model's predictive performance. R^2cv closer to 1.0 indicates stronger predictive power. For a reliable model, R2cv should be reasonably close to the R^2 value obtained during training. The formula for R^2cv is shown in Equation (4)

$$R^2 = 1 - \frac{\sum_{i=1}^{n} (\acute{y}_i - y_i)^2}{\sum_{i=1}^{n} (y_i - \bar{y})^2} \quad (2)$$

$$RMSE = \sqrt{\frac{1}{n}\sum_{i=1}^{n} (y_i - \acute{y}_i)^2} \quad (3)$$

$$R^2cv = 1 - \frac{\sum_{i=1}^{n} (y_i - \acute{y}_i cv)^2}{\sum_{i=1}^{n} (y_i - \bar{y})^2} \quad (4)$$

Where n represents the number of compounds used in the model prediction; y_i and $\acute{y}_i$ represent the actual and predicted pK_a values of the ith compound, respectively; and $\bar{y}$ represents the average of the actual pK_a values of the n predicted compound.

Table S2. Hyperparameters to be tested for RF, ANN, and XGB.

**Random forest (RF)**		**Artificial Neural Network (ANN)**		**Extreme Gradient Boosting (XGB)**	
**Hyperparameter**	**Value tested**	**Hyperparameter**	**Value tested**	**Hyperparameter**	**Value tested**
max feature	2	hidden layer	(5,2)	n estimators	350
min samples leaf	1	batch size	1	max depth	10
min samples split	2	learning rate	0.001	learning rate	0.01
n estimators	70			min child weight	2

Table S3. Experimental and predicted pK_a of 56 amino lipids of three models using GFA descriptor selection method.

**Code**	**Exp. pK_a**	**pK_a prediction**					
		**RF**	**Residual**	**ANN**	**Residual**	**XGB**	**Residual**
**A01***	6.680	6.611	0.069	6.341	0.339	6.699	-0.019
**A02**	5.970	6.279	-0.309	6.145	-0.175	6.202	-0.232
**A03**	5.940	5.933	0.007	5.672	0.268	5.829	0.111
**A04**	6.650	6.611	0.039	6.341	0.309	6.699	-0.049
**A05**	6.790	6.611	0.179	6.341	0.449	6.699	0.091
**A06***	6.420	6.512	-0.092	6.711	-0.291	6.425	-0.005
**A07**	6.430	6.512	-0.082	6.711	-0.281	6.425	0.005
**A08***	7.290	6.312	0.978				
